# A systematic review and meta-analysis of interventions to decrease cyberbullying perpetration and victimization: An in-depth analysis within the Asia Pacific region

**DOI:** 10.3389/fpsyt.2023.1014258

**Published:** 2023-01-27

**Authors:** Ida Khairina Kamaruddin, Aini Marina Ma’rof, Ahmad Iqmer Nashriq Mohd Nazan, Habibah Ab Jalil

**Affiliations:** ^1^Faculty of Educational Studies, Universiti Putra Malaysia, Serdang, Malaysia; ^2^Faculty of Medicine and Health Sciences, Universiti Putra Malaysia, Serdang, Malaysia

**Keywords:** cyberbullying perpetration, cyberbullying victimization, intervention, systematic review and meta-analysis, Asia-Pacific

## Abstract

**Background:**

Cyberbullying perpetration and victimization are prevalent issues in adolescent development and are a rising public health concern. Numerous interventions have been developed and implemented to decrease cyberbullying perpetration and victimization. Through an updated systematic review and meta-analysis, this study aimed to tackle a significant gap in the cyberbullying literature by addressing the need to empirically determine the effectiveness of programs with non-school-aged samples with a specific focus on studies conducted within the Asia-Pacific region.

**Methods:**

A systematic literature review was conducted to identify intervention research to reduce cyberbullying perpetration and victimization published from January 1995 to February 2022. Ten electronic databases—Cambridge Journal Online, EBSCOHOST, ERIC, IEEE XPLORE, Oxford Journal Online, ProQuest Dissertations and Theses, PubMed (Medline), Science Direct, Scopus, Springerlink—and a subsequent manual search were conducted. Detailed information was extracted, including the summary data that could be used to estimate effect sizes. The studies’ methodological quality was assessed using the Effective Public Health Practice Project (EPHPP) quality assessment tool.

**Findings:**

Eleven studies were included in the review of the 2,540 studies identified through databases, and 114 additional records were discovered through citation searching. Only four studies were included in the meta-analysis, exploring game-based, skill-building, school-based, and whole-school interventions. The first meta-analysis pooled estimates from these four studies that assessed cyberbullying perpetration frequency using continuous data post-intervention. These studies reported data from 3,273 participants (intervention *n* = 1,802 and control *n* = 1,471). A small but not statistically significant improvement favoring the intervention group from pre- to post-intervention was shown by the pooled effect size, −0.04 (95% CI [−0.10,0.03], *Z* = 1.11, *P* = 0.27). The second meta-analysis included two qualified studies investigating cyberbullying victimization frequency using continuous data at post-intervention among 2,954 participants (intervention *n* = 1,623 and control *n* = 1,331). A very small but non-significant effect favoring the intervention group was discovered.

**Conclusion:**

This research primarily highlights that the endeavor for cyberbullying intervention is still developing in the Asia-Pacific region, currently involving a limited set of stakeholders, settings, and delivery modes. Overall, meta-analyses of cyberbullying interventions conducted in the Asia Pacific found no significant effects in reducing cyberbullying perpetration and victimization.

## 1. Introduction

Surveys have revealed that 93% of teenagers owned smartphones and mobile devices were used by more than 90% of them to access online information before the COVID-19 outbreak (1). Technology will remain an essential part of their lives throughout the pandemic as well as after it (2). This heavy reliance on technology has resulted in an increase in cyberbullying. Cyberbullying perpetration is the act of sending, posting, or sharing negative, harmful, false, or mean content about someone else through various forms of digital technology (3). Cyberbullying has affected ∼14–21% of youths over the past decade (either as a victim, a bully, or a bully-victim) (4–6). According to research conducted in the US, ∼15% of students have experienced or perpetrated cyberbullying in the past 30 days (7). In other countries varying prevalence rates were reported, such as Australia at 5.0% and Canada at 23.8% (8). It appears that cyberbullying increased during the epidemic, perhaps due to students’ intensive use of technology (9).

Over the past 15 years, 50 studies have evaluated the effectiveness of cyberbullying interventions, as reported in a comprehensive systematic review and meta-analysis by Polanin et al. (2). They extracted 320 total effect sizes from these primary studies, covering over 45,000 participants and several continents. Overall, it is estimated that the programs included in the synthesis could reduce cyberbullying perpetration by 76% and cyberbullying victimization by 73%. Among these studies, the skill-building component was included in almost 80% of the programs, and many others used curricula and prepared materials, psychoeducation, or multimedia. The modality varies greatly among interventions that were found to be effective at reducing cyberbullying perpetration and victimization. For example, online instruction was used in the Non-cadiamointrappola program in Italy (10) to deliver content and create interactive experiences for students that extend beyond the classroom. Skills for Life (11) is another illustration of an effective intervention program that builds on rational-emotive behavior therapy and social learning theory to improve social, emotional, and moral skills. This program was integrated into the schools’ curriculum in the Netherlands for two academic years, using techniques like role-playing, discussion, and modeling with video extracts. As previously found with other social-emotional learning programs, this intervention can have a positive impact on many health outcomes, particularly for disadvantaged students. Such research findings would provide important insight into cyberbullying issues for future researchers, program developers, educators, and policymakers.

Although most cyberbullying studies have been conducted in the US and Western countries, the burden Malaysia and many other Asia Pacific countries face are comparable to that experienced by Americans or Europeans. In a global survey by IPSOS, Malaysia ranks third after South Africa and Peru, with 34% reporting knowing a child who has been cyberbullied (12). According to this report, the majority of cyberbullying among children in the Asia Pacific region is perpetrated by a victim’s classmate or known individuals. Social networking sites are the most common source of cyberbullying for children in Asia Pacific countries (53%), followed by online messaging (48%) and mobile devices (46%). Consistent with 2019 data from UNICEF (13) on young people in 30 countries, this report also found that 33% of parents are aware of a child being cyberbullied in their community. This report significantly revealed that parents around the world, including those in the Asia Pacific region, are reporting an increase in the prevalence of cyberbullying among their children.

### 1.1. Rationale/Significance of research

Understanding the implications of research on cyberbullying for prevention and intervention programs is crucial for relevant government bodies seeking to deter those aggressive behaviors. Empirical findings on cyberbullying interventions can help policymakers and professionals understand precisely how to combat the negative cyber-bully/victim impact. Thus, the current study aimed to identify scholarly efforts across contexts necessary to advance anti-bullying programs, especially in the Asia Pacific region. Essential for the continual progress of program development, the results of this study will be helpful to professionals across various disciplines to be better informed of not only what is happening globally but regionally and locally, as well as to have more meaningful and extensive empirical findings to sample and make decisions from.

With the growing incidence of cyberbullying around the world, researchers, practitioners, and politicians are collaborating to eradicate this particularly damaging type of violence. Due to the severe consequences and rising prevalence of cyberbullying worldwide, this trend has drawn more and more attention. However, few studies on cyberbullying have been conducted in Asia Pacific nations, compared to the number of studies conducted in Western nations. This is especially evident in a recent global systematic review by Zhu et al. (14), who found only thirteen studies (out of 63) since 2015 examining cyberbullying among children and adolescents in Asia Pacific countries. China has the highest prevalence of cyberbullying perpetration (46%), as reported by Lin (15), while research from Canada (16) and South Korea (17) found that these nations had the lowest prevalences of cyberbullying perpetration (8 and 6%). Spain had the highest prevalence of victims of cyberbullying (58%) (18), followed by Malaysia (52%) (19), Israel (45%) (20), and China (45%) (21). The countries with the lowest reported victim rates were Canada (14%) (16) and South Korea (15%) (17). With the growing incidence of cyberbullying worldwide, researchers, practitioners, and politicians should collaborate to eradicate this particularly damaging type of violence. Even though children and youth are using electronic media more frequently than ever, cyberbullying is still an understudied problem in the Asia-Pacific region, where it is likely to be an equally important issue.

In light of the emerging evidence, researchers have attempted to synthesize the literature available regarding the effects of anti-cyberbullying programs. Existing interventions either specifically target cyberbullying or generally address it in bullying or school violence prevention programs. The existing reviews differ from this current review by being out of date (22) or lacking the use of modern meta-analytic techniques (23). In addition, several researchers have conducted reviews on cyberbullying programs’ effects. However, these reviews synthesized correlation or prevalence effect sizes and therefore do not provide evidence of program effectiveness (7, 24–27). The most comprehensive and up-to-date review using advanced meta-analytical techniques was conducted by Polanin et al. (2). However, the study falls short of highlighting the specific components of interventions that are effective, especially the effectiveness of programs with non-school-aged samples with a specific focus on studies conducted within the Asia-Pacific region. A summary of all these reviews can be found in Table 1.

**TABLE 1:** Summary of previous systematic reviews and meta-analysis on cyberbullying.

References	Total studies			Year of publication	Objectives	Findings
	**Identified**	SR*	MA**			
Mishna et al. (22)	3,029	3	–	2003–2006	To systematically review the effectiveness of cyber abuse interventions in increasing Internet safety knowledge and decreasing risky online behavior.	Participation in cyber abuse prevention and intervention strategies is associated with an increase in Internet safety knowledge.
Gaffney et al. (23)	3,994	24	18	2012–2018	To evaluate the effectiveness of cyberbullying intervention and prevention programs implemented with school-age children.	Anti-cyberbullying programs can reduce cyberbullying perpetration by approximately 10–15% and cyberbullying victimization by approximately 14%.
Gardella et al. (24)	9,312	12	9	2009–2014	To quantitatively synthesize relationships between Peer cyber-victimization (PCV) and educational outcomes.	PCV is associated with higher school attendance problems and academic achievement problems.
Guo (25)	479	77	77	2004–2013	To determine the target factors predicting individuals’ perpetration and victimization in cyberbullying.	A prior history of bullying others offline and committing problem behaviors were the two strongest predictors of cyberbullying perpetration. Long-term psychological problems and previous offline victimization were significant predictors of cyberbullying victimization.
Marciano et al. (26)	3,613	56	56	2007–2017	To conduct a meta-analysis quantitatively summarizing exclusively longitudinal studies on the causes and consequences of cyberbullying perpetration (CP) and victimization (CV).	CP and CV have significant effects on internalizing problems, externalizing problem behaviors, and peer relations. CV has a greater impact on older adolescents and females whereas older men are more likely to be cyberbullies.
Modecki et al. (7)	1,951	80	80	2006–2013	To conduct a thorough review of the literature and identify studies that reported corresponding prevalence rates for cyber and traditional bullying and/or aggression in adolescents.	The prevalence of cyberbullying was lower than that of traditional bullying, and the two were highly correlated.
Zych et al. (27)	1,545	66	–	2007–2015	To conduct a systematic review of systematic reviews and meta-analyses of research about bullying and cyberbullying.	Anti-bullying interventions might be effective in reducing bullying, although the effect sizes are small and depend on the components of the programs.
Polanin et al. (2)	11,588	50	50	2004–2019	To conduct a systematic review and meta-analysis that synthesized the effects of school-based programs on cyberbullying perpetration or victimization outcomes.	The effectiveness of the prevention programs was found for both perpetration and victimization of cyberbullying, with a slightly higher effect size for perpetration over victimization.

*Systematic review, **meta-analysis.

Studying the effectiveness of available cyberbullying interventions in the context of the Asia-Pacific framework is pertinent, where pressure based on collectivistic ideals and rigid cultural scripts for social interactions remains strong among the majority of Asia-Pacific countries as compared to Western cultures (28). Group-focused values and behaviors emphasizing on maintaining group relations, social conformity, and avoiding interpersonal conflict can plausibly influence cyberbullying behaviors in this region bidirectionally. Strong social norms can lead to lower tolerance for deviant behaviors among group members, resulting in lower involvement in bullying incidences compared to Western countries (29, 30). However, strong social norms and collectivistic values may also lead to high conformity to group behaviors and could impact the prevalence and severity of cyberbullying behaviors within the Asia-Pacific region. Collectivistic adolescents may be more likely to cyberbully others as a way of conforming to the group norm or for penalizing someone who does not adhere to such collectivistic ideals (31).

Factors such as gender socialization experience, parent-child relationships, and cultural norms in most Asia-Pacific countries also differ from the West and have been implicated to influence cyberbullying incidences in this region (30). These socio-cultural factors have also been known to vary somewhat even among the countries (32, 33). These unique contributing factors highlight the unique mechanisms through which cyberbullying operates which in turn may lead to unique ways on how cyberbullying interventions are developed and implemented in this region compared to the West.

Given the wide-ranging and pervasive problems caused by cyberbullying, the extensive resources devoted to it, and the lack of a comprehensive and up-to-date meta-analytic review of programs to prevent it within the Asia Pacific literature, an updated systematic review and meta-analysis synthesizing the effects of programs on cyberbullying perpetration or victimization outcomes is warranted.

### 1.2. Objectives

This study seeks to comprehensively analyze studies examining interventions’ effects on cyberbullying perpetration and victimization outcomes. Despite a number of extensive systematic reviews and meta-analyses [e.g., (2, 34, 35)], we consider it is vital to resynthesize the various primary research findings to provide a concrete and appropriate response to cyber violence in policy and practice, particularly in Asia Pacific region. To address the research gap within the Asia-Pacific region on online user rights and protection concerning cyberbullying victims and perpetrators, this study aimed to provide further valuable empirical evidence by extending the work of the most recent large-scale systematic review and meta-analysis study on interventions to decrease cyberbullying perpetration and victimization (2, 36) by expanding the age-range beyond school-aged settings. Specifically, this research sought to conduct an updated systematic review on intervention effects to decrease cyberbullying perpetration and victimization by considering literature within the Asia-Pacific region, which was not the focus covered by previous credible reviews.

## 2. Methods

This systematic review and meta-analysis study was carried out following PRISMA 2020 guidelines to support quality and dependability (37).

### 2.1. Data collection

#### 2.1.1. Inclusion/Exclusion criteria

Population: We expanded eligible studies beyond the K-12 age group [i.e., Kindergarten (5–6-year-olds) until upper secondary six and equivalent (17–18-year-olds)] to include non-school children.

**Intervention Studies.** Studies on interventions designed to reduce cyberbullying perpetration and victimization were included in this review, regardless of the type of intervention. This gave us a wide range of studies to draw from, including those focusing on direct interventions, as well as those exploring broader violence prevention initiatives and anti-bullying programs.

**Comparison Group.** For the study to be considered for the review, it was required to contain a comparison group that met specific eligibility criteria. The comparison group could have been composed of individuals who did not receive any form of intervention, those who underwent treatment as per usual practice, or those who received a treatment that was either minimal or demonstrated to be ineffective. The comparison group was necessary to provide a point of reference for evaluating the effectiveness of the intervention being studied. Without such a group, it would be difficult to draw any meaningful conclusions about the efficacy of the intervention in question.

**Research Design.** We included randomized controlled trials, quasi-experimental studies, and studies that may have assigned groups in a randomized or non-randomized way to conditions without any exclusion based on the level of assignment.

**Primary Outcome Measures.** This review included studies that assessed cyberbullying perpetration or victimization as the primary outcome measure and did not exclude those that utilized a broader program to prevent violence or bullying instead of a specific cyberbullying intervention. This procedure and the reasoning behind it have been explained by Polanin et al. (38). The exclusion of certain studies was found to alter significant conclusions in their meta-analysis. Another rationale for this is previous meta-analytic studies’ finding that the perpetration and victimization of conventional bullying and cyberbullying are connected (26).

**Time range.** Although cyberbullying-related terms started appearing in literature around 2003, studies published since 1995 were also included to ensure comprehensive coverage of research.

**Publication Status.** To minimize publication bias, we searched for relevant information on cyberbullying, including published and unpublished research reports and available data sets (39) with cyberbullying perpetration and victimization measures.

**Language.** Publications must be in English or Bahasa Melayu, regardless of the country of origin, were included in our review.

### 2.2. Literature search and screening

We employed multiple methods to identify qualifying studies, including electronic bibliographic searches and forward and backward reference harvesting. Our search included published and unpublished works within the traditional and gray literature. We used tailored search terms for each database and the following online databases available through our University’s library services: Cambridge Journal Online, EBSCOhost, ERIC, IEEE XPLORE, Oxford Journal Online, ProQuest Dissertations and Theses, PubMed (Medline), Science Direct, Scopus, and SpringerLink. The literature search summary is included in Supplementary Data Sheet 1, and detailed records are presented in Supplementary Data Sheet 4. We finalized the search key terms (see Supplementary Data Sheet 3) and applied those to several search strategies for each database.

### 2.3. PRISMA flowchart

#### 2.3.1. Abstract screening

We employed a comprehensive approach to review the numerous studies located during this round of research (as outlined in 2 and 36). All members of the review team assessed the abstracts. We developed a screening guide for abstracts (see Supplementary material) and utilized the free Rayyan software (40) for web-based abstract screening.

#### 2.3.2. Full-article retrieval

To prepare for the next screening stage, the team members retrieved the complete article PDFs of the previously screened titles and abstracts.

#### 2.3.3. Full-article screening

The team carried out a thorough screening process for eligibility by entering responses into a designated tool, followed by review and validation by the principal investigator and lead statistician, and after a training session, a pilot screening was conducted.

#### 2.3.4. Data Extraction

We created a codebook to document all data that was extracted from each study. The data comprised demographics of the sample, characteristics of the intervention and comparison conditions, and summary statistics useful for effect size estimation. An Excel-based relational database was designed to structure the information. To maintain accuracy and consistency, coders used dedicated coding screens in Excel for each category of extracted data.

### 2.4. Data analysis

We conducted separate analyses for each outcome variable category: (1) cyberbullying perpetration and (2) cyberbullying victimization. The characteristics of the studies included in the analysis were documented, including the publication status, the target of the program, the type of research design, and geographical location. We also planned to perform a sub-analysis looking further into the potential differentiated effects of gender, randomized controlled trial vs. non-randomized control trial designs, whether or not the studies were theory-based or non-theory-based, and geographical locations with a specific focus on Asia-Pacific regions, and studies that also covers the age-range beyond K-12.

### 2.4.1. Meta-analyses

We analyzed two primary outcome variables using meta-analytic models, with separate analyses using a random-effects model and robust variance estimation method (41). The random-effects model considered both within-study and between-study variations, making it suitable for studies with diverse populations and designs. The robust variance estimation method was used to estimate standard errors of effect size estimates and to adjust for potential biases due to small sample sizes or heterogeneity. Each effect size estimate was weighted by its inverse variance to calculate the average effect size (42). The model assigned greater importance to effect sizes with smaller variances, resulting in a more accurate estimation of the overall effect size.

Our original plan, which followed the preceding step, involved conducting two confirmatory meta-regression analyses to investigate the potential predictors contributing to cyberbullying perpetration and victimization. Our meta-regression approach would involve incorporating several pertinent variables. These variables would encompass the type of effect size, the objectives of the intervention, the native country of the study participants, the timing of the follow-up measurement, as well as the percentage of male participants and individuals from ethnic minority groups. Sub-analyses were also planned to examine potential moderating effects of gender, study design, theory-based or non-theory-based, geographical location, and age range. Sub-analyses would be conducted to evaluate the potential moderating effects of gender, comparing randomized controlled trial study designs vs. non-randomized control trial designs, whether or not the studies were theory-based or non-theory-based, taking geographical locations of subjects into consideration, and expanding the age range beyond school-aged settings.

### 2.4.2. Exploratory analysis

Ultimately, we performed an exploratory analysis aimed at assessing the overall effect size of the specified interventions that were identified during our review process.

## 3. Findings

### 3.1. Search outcomes

**FIGURE 1:**
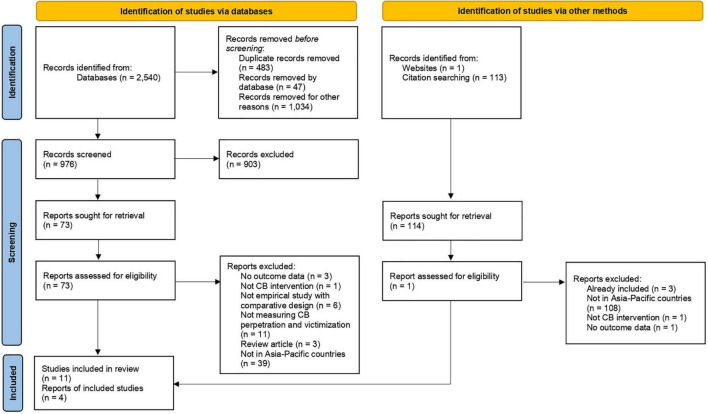
PRISMA 2020 flow diagram.

In this review, we identified all publications that reported the effectiveness of cyberbullying interventions after 1995 based on the PRISMA guidelines. Countries included were Australia, Brunei, Myanmar (Burma), Cambodia, China, Fiji, Indonesia, Japan, Kiribati, Laos, Malaysia, Marshall Islands, Micronesia, Mongolia, Nauru, New Zealand, North Korea, Palau, Papua New Guinea, Philippines, Samoa, Singapore, Solomon Islands, South Korea, Taiwan, Thailand, Timor-Leste, Tonga, Tuvalu, Vanuatu, and Vietnam. The search yielded 2,540 studies, with 114 additional records identified through citation searching (*n* = 113) and websites (*n* = 1). After removing duplicates and records based on their titles, 976 records were left for abstract screening. A total of 903 abstracts were excluded for failing to meet one or more inclusion criteria during this screening process. The remaining 73 studies were reviewed as full text (see Table 2 and Supplementary Data Sheet 5). Of these, 63 more studies were excluded, leaving 11 relevant records (ten *via* database and one *via* other methods) to be included in this review. We evaluated four studies further through meta-analysis. A PRISMA Flow Diagram can be found in Figure 1, which details the full results of our search, screening process, and reasons for the exclusion of studies.

**TABLE 2:** Summary of studies included in the systematic review.

References	Study design	Intervention components	Target group	Implementation methods	Activities	Primary outcome	Quality of study (A-F)
Tapingkae et al. (48)	Quasi-design	Formative assessment-based contextual digital gaming approach as the in-class learning activity to teach digital citizenship	*N* = 115; 12—14-year-old students in Thailand	Digital game-based learning	Gaming scenario	Digital citizenship behaviors Online harassment victimization Online harassment perpetration Learning motivation Learning perception	SB: 1 D: 1 C: 1 B: 2 DCM: 1 W&DO: 1 Global rating: **High quality**
Leung et al. (49)	RCT	Attitudes about cyberbullying behavior and increase their awareness of cyberbullying	*N* = 137; 19–28-year-old college students in Hong Kong	Skill-building	Role-play activity, video, group discussion, self-reflection writing task	Awareness of cyberbullying Attitude toward cyberbullying	SB: 1 D: 1 C: 1 B: 2 DCM: 1 W&DO: 2 Global rating: **High quality**
Liau et al. (45)	Quasi-design	Hands-on digital exhibition involving peer-mentoring and a transmedia adventure storytelling mode within a multisystemic approach	*N* = 440; 8–11-year-old elementary school students in Singapore	School-based intervention	Peer mentoring, digital exhibition	Attitudes toward risky online behaviors Cyberbullying and offline meeting Mentees’ perceptions of their mentors Mentors’ perceptions of their mentoring experience	SB: 2 D: 2 C: 3 B: 2 DCM: 1 W&DO: 1 Global rating: **Moderate**
Cross et al. (47)	c-RCT	Whole-school and student-level resources and training	*N* = 3,382; 13-year-old secondary school students in Perth, Australia	A whole-school program to enhance the capacity of school staff, students, and families to respond effectively to reduce cyberbullying behavior	Socio-ecological program assisting staff in implementing strategies related to their school’s organizational context	Cyberbullying victimization and perpetration behavior	SB: 1 D: 1 C: 1 B: 2 DCM: 1 W&DO: 1 Global rating: **High quality**
Lee et al. (67)	Quasi-design	WebQuest cyberbullying prevention course	*N* = 61; Junior high school students in Taiwan	Cyberbullying prevention WebQuest course	A set of student-centered and exploration-oriented learning activities presented in a webpage layout	Knowledge about cyberbullying Attitude toward cyberbullying Cyberbullying intentions	SB: 1 D: 2 C: 1 B: 2 DCM: 1 W&DO: 1 Global rating: **High quality**
Ng et al. (44)	RCT	Brief mindfulness practice as an intervention on the relationship between cyberbullying and depressive symptoms	*N* = 82; 19–28-year-old young adults in Malaysia	Brief mindfulness practice (STOP)	Video	Cyberbullying victimization Mindfulness level	SB: 3 D: 1 C: 3 B: 1 DCM: 1 W&DO: 2 Global rating: **Low quality**
Cross et al. (47)	c-RCT	Individualized training and resources to support students’ transition and reduce bullying	*N* = 3,769; Aged 13 secondary school students in Perth, Australia	Friendly schools whole-school curriculum modules	Training and coaching support	Victimization and perpetration Loneliness Safety Mental wellbeing	SB: 1 D: 1 C: 1 B: 2 DCM: 1 W&DO: 1 Global rating: **High quality**
Cross et al. (47)	c-RCT	Multidimensional school-based programs with strategies targeting all levels of the school community	*N* = 3,382; Aged 13 secondary school students in Perth, Australia	Cyber Friendly Schools Project (CFSP) whole-school curriculum modules	Teaching and learning resources and a website resource	Cyberbullying victimization and perpetration behavior	SB: 1 D: 1 C: 1 B: 2 DCM: 1 W&DO: 1 Global rating: **High quality**
Peng et al. (65)	Quasi-design	Educational intervention based on the knowledge-attitude-practice model	*N* = 328; Junior high school students in Shantou, China	Raising students’ awareness of school bullying through educational intervention	Bullying-themed class meetings, distributing bullying educational leaflets at school and playing anti-bullying videos in class	Awareness of bullying and acceptance of school anti-bullying education Peer victimization and bullying Cyber victimization and cyberbullying	SB: 1 D: 2 C: 1 B: 2 DCM: 1 W&DO: 1 Global rating: **High quality**
Wiretna et al. (46)	Quasi-design	Solution-focused brief counseling (SFBC) to reduce student online aggression behavior	*N* = 12; High school students with high online aggression in Yogyakarta, Indonesia	Counseling	Counselee finding solutions to the counseling process	Online aggression	SB: 3 D: 2 C: 1 B: 2 DCM: 1 W&DO: 1 Global rating: **Moderate**
Leung et al. (49)	RCT	E-course on cyberbullying	*N* = 144; 19–23-year-old undergraduate students in Hong Kong	Anti-cyberbullying online classes	Interactive course materials, including computer-simulated scenarios, popular Internet incidents, and role-play games	Time spent on social media Past involvement in CB Awareness of CB Intention to help CB victims Perceived behavioral control about helping CB victims Self-efficacy to combat CB Likelihood of behavioral intervention in CB	SB: 1 D: 1 C: 1 B: 2 DCM: 1 W&DO: 1 Global rating: **High quality**

### 3.2. Study characteristics

Table 2 provides an overview of the studies that were included. Study design (RCT, c-RCT, and quasi), intervention components (e.g., digital exhibition and cyberbullying prevention course), target group (sample’s size, age, and country where the study was conducted), implementation methods (e.g., school-based and game-based), activities related to the various program elements (e.g., gaming scenario, role-play, and video), primary outcomes (e.g., cyberbullying victimization and perpetration behavior), and quality of the study (low, moderate, and high) were extracted from each study. The primary outcomes were reported using self-report data in each study, but the measures used to record them varied. Out of the 11 studies reviewed, there were three randomized controlled trials (RCTs), three cluster randomized controlled trials (c-RCTs), and five quasi-experiments. These studies were published between 2013 and 2022. The sample’s age range was 8 to 29 years old, and the number of participants ranged from 12 to 3,769. Almost all of the studies (9 out of 11) focused on school-aged children, with only two studies conducted on non-school children (aged 19 to 28 years). Study participants covered in this review were strictly enrolled from the Asia-Pacific countries. Specifically, the studies included were conducted in Australia (*n* = 3), Hong Kong (*n* = 2), Thailand (*n* = 1), Singapore (*n* = 1), China (*n* = 1), Taiwan (*n* = 1), Indonesia (*n* = 1), and Malaysia (*n* = 1). Intervention programs in these eleven studies took anywhere between 1 day and 3 years to fully implement.

### 3.3. Study quality assessment

The methodological quality of the studies is presented in the final column of Table 2, which was completed using the Effective Public Health Practice Project checklist (EPHPP, (43) tool. At this stage, two reviewers discussed with each other to reach a consensus in case of disagreements on study quality or data extraction. Based on the EPHPP Quality Assessment Tool and its dictionary guidelines, the included studies were rated on a scale of 1 (strong), 2 (moderate), or 3 (weak) for each category accordingly. These categories include selection bias (SB), study design (D), confounders (C), blinding (B), data collection method (DCM), withdrawal and dropouts (W&DO), and overall quality (global rating). A global rating of low, moderate, and high was determined by averaging these six categories’ rankings. Studies without any weak ratings across all categories were rated as having a strong level of quality in their final global rating. Studies of a moderately strong quality have one category rated as weak, while those rated as qualitatively weak have a weak rating in two or more categories. The subcategory “data collection method” (DCM) was rated as strong in all studies, while the subcategory “withdrawal and dropouts” (W&DO) had only two studies evaluated as moderate, and the subcategory “design” (D) had four studies evaluated as moderate. Six (*n* = 6) of the included studies were well-designed RCTs and c-RCTs, which provided detailed descriptions of the methods used and were assessed to be at low risk of bias. The two weakest subcategories were the “selection bias” (SB) and “confounders” (C), with one study evaluated as moderate and two studies evaluated as weak for both. Finally, except for one study evaluated as strong (44), the risk of bias was deemed moderate under the “blinding” (B) subcategory for all studies due to the absence of explicit information detailing the assignment of study participants to delivery strategies. Reports of study participants’ different characteristics at baseline were noted in all studies, which could minimize potentially additional sources of bias. For global rating, eight studies were classified as “high quality,” while two others were rated as “moderate” (45, 46). Only one study (44) was considered to have “low quality” and could not be considered for inclusion in the meta-analysis.

### 3.4. Studies included in meta-analysis

Four out of the eleven studies reviewed qualified for meta-analyses. Among the four studies eligible for inclusion, there was one c-RCT study (47), two quasi-experiments (45, 48), and one RCT (49). Study participants were mainly from secondary schools (47, 48), elementary schools (45), and college students (49). In terms of the percentage of girls participating, Leung et al. (49) included 76%, and Liau et al. (45) had 48%, while the other two studies did not mention gender explicitly. A school-based intervention was developed and implemented in Singapore and Australia by Liau et al. (45) and Cross et al. (47), respectively. Meanwhile, Tapingkae et al. (48) implemented a digital game-based learning intervention in Thailand, while Leung et al. (49) developed a skill-building intervention in Hong Kong. A range of intervention techniques was used in these four studies, including training, role-playing, group discussions, gaming scenarios, and peer mentoring. Various lengths of time were allotted for the program, ranging from 1 day to three school years. Other than Cross et al. (47), who did not provide information on the duration of the intervention session, the rest of the studies reported sessions between 30 and 45 min. Detailed study characteristics are presented in Table 2 (studies included in the meta-analysis are highlighted in gray), and their summary statistics are shown in Table 3.

**TABLE 3:** Summary statistics of cyberbullying interventions on cyberbullying perpetration.

References	Experimental *M* (*SD*)	Control *M* (*SD*)	Interpretation of the outcome
Liau et al. (45)	0.20 (0.44) 136	0.23 (0.49) 101	The higher the score, the higher the agreement with the online risk behavior (ORB)
Cross et al. (47)	0.03 (0.22) 1,538	0.03 (0.25) 1,246	The higher the score, the higher the cyberbullying experience
Tapingkae et al. (48)	0.14 (0.26) 60	0.26 (0.36) 55	The higher the score, the higher the online harassment perpetration behavior
Leung et al. (49)	2.13 (0.85) 68	2.27 (0.85) 69	The higher the score, the higher the positive attitude toward cyberbullying

### 3.5. Meta-analysis results

In this review, we considered that any amount of statistical heterogeneity would be acceptable, and any estimates of the average effect of intervention were worth reporting. Statistical heterogeneity of the included studies was explored using the *I*^2^ statistics, while we assessed the risk of bias based on the EPHPP criteria. We used ReviewManager (RevMan 5.4) built-in variance correction to calculate 95% confidence intervals to reflect the uncertainty in heterogeneity estimates. Analysis was also carried out using the random effects option within the RevMan program to report odds ratios.

**FIGURE 2:**
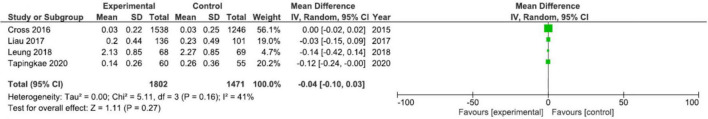
Forest plot of cyberbullying perpetration frequency at post-intervention among four included studies reporting continuous data.

We conducted two separate meta-analyses and a synthesis of effect sizes following their consistency in the types of interventions, study designs, and outcome variables. The first meta-analysis pooled estimates from four studies (*n* = 4) that assessed cyberbullying perpetration frequency using continuous data post-intervention. These studies reported data from 3,273 participants (intervention *n* = 1,802 and control *n* = 1,471). We found low heterogeneity between the studies *Tau*^2^ = 0.00(χ^2^ = 5.11,*df* = 3,*P* = 0.16)and *I*^2^ = 41%. Our findings found that the resulting pooled effect size was −0.04(95%*CI*[−0.10,0.03],*Z* = 1.11,*P* = 0.27), indicating a small but non-significant improvement favoring the intervention group from pre-intervention to post-intervention (see Figure 2).

**FIGURE 3:**
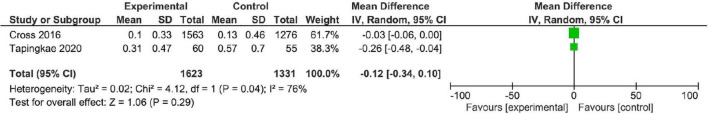
Forest plot of cyberbullying victimization frequency at post-intervention among two included studies reporting continuous data.

The second meta-analysis included two studies investigating cyberbullying victimization frequency using continuous data at post-intervention among 2,954 participants (intervention *n* = 1,623 and control *n* = 1,331). We found a very small, but no significant effect, favoring the intervention group (*MD* = −0.12, 95%*CI*[−0.34,0.10],*Z* = 1.06,*P* = 0.29) with significant substantial heterogeneity (*I*^2^ = 76%,*P* = .04) (see Figure 3). This substantial variability appeared due to the small number of studies included in the analysis rather than sampling error (50).

Subgroup analyses for study design, theory application, and intervention setting were not performed, given the nature of the studies included in the meta-analyses. A minimum of two studies are required to conduct any subgroup assessments; however, only Cross et al. (47) adopted the randomized controlled trial study design, while Leung et al. (49) was the only study conducted outside the school setting focusing on college students instead. Cross et al. (47) was also the sole study that implemented a theory-based intervention. Nonetheless, it is worth noting that Liau et al. (45) utilized the Theory of Planned Behavior to measure their primary outcome (i.e., attitude), but that same theory was not part of the cyber wellness intervention development.

## 4. Discussion

This study identified published literature on cyberbullying intervention aimed at reducing cyberbullying perpetration and cyberbullying victimization. Even though there are limitations due to the small number of available studies, we believe this report addresses a critical gap in the cyberbullying literature by demonstrating the current state of cyberbullying interventions in the Asia-Pacific region. The present meta-analysis showed, on average, a small but non-significant reduction in cyberbullying perpetration and victimization. The small cumulative effect observed in this study could be attributed to the short intervention period of the included studies (i.e., in the range of 1 day up to three school years). A review of evaluation research on bullying suggested that intervention should last up to 2 years before substantial change can be seen in the outcomes being assessed (51), as the frequency of reported cyberbullying behavior might have been low within a period of one or two school semesters (possibly three to four events within the 10–12 weeks period) (47). Hence, the room to shift the frequency or severity was limited. Additionally, despite being universal programs, whole-school programs appear to be less effective at combating bullying perpetrators, perhaps because only 10–20% of students are involved in bullying behaviors (52).

This is the first study to investigate the effectiveness of cyberbullying interventions in Asia-Pacific countries. This research primarily highlights that the endeavor for cyberbullying intervention is still developing in the Asia-Pacific region, currently involving a limited set of stakeholders, settings, and delivery modes. The low heterogeneity in our meta-analysis of cyberbullying perpetration suggested that the studies, target populations, and interventions were most likely highly comparable. Overall, meta-analyses of cyberbullying interventions conducted in the Asia Pacific found no significant effects in reducing cyberbullying perpetration and victimization.

### 4.1. Future research

With the nature of the world wide web or internet transformation and the addition of new technologies such as virtual reality, little is known regarding the nature of people’s interaction and its evolution with these technological advancements. With ever-increasing channels for interaction, it is critical to understand its impact on young people, who are also the fastest adopters of new ways of interaction and technology. For example, online interaction has now evolved into online virtual spaces, i.e., the metaverse (53). With the world facing many challenges from the 2020 pandemic, online education has also become a norm, further legitimizing our youths’ increased screen time (54, 55). However, the overall impact of a significant increase in screen time usage has yet to be thoroughly studied and reported in academic literature. The interventions suggested by the studies in this report seem to view and address the problem of cyberbullying as static. Given the extensive use of the Internet and social media by children and youths, research should address the dynamic nature of these social-online interactions, as various forms of bullying continue to evolve and expand in tandem with the number of ways and mediums of social interaction. Future studies should address cyberbullying as a continuously evolving problem and find ways to address this dynamic problem coupled with the ever-increasing pace of technology. In order to understand cyberbullying more thoroughly, future studies must also investigate the new ways in which humans interact as well as the technologies that enable it.

Educational institutions should embed cyber awareness and media literacy in existing subjects, taught implicitly throughout classroom practices. Teachers should integrate media literacy into their instructional strategies rather than teaching it as a separate subject. This effort increases exposure, models multiple uses for media literacy in various contexts, and reduces the need for extra subjects in already packed schedules. For these reasons, it is more effective to integrate media literacy education than to treat it as an isolated subject (56). Future studies are therefore recommended to design interventions that indirectly target cyber awareness and media literacy in existing classroom instruction.

The studies reviewed in this report also lack guidance from essential theories (i.e., developmental, organizational, socio-emotional, socio-cultural, social-cognition, peer response, dominance, and humiliation theories). This caveat resulted in non-significant and smaller effect sizes, limiting the findings’ application in the real world. For example, the studies covering interventions in Asia-Pacific regions did not target the area of participants’ socio-emotional skills explicitly. However, extensive empirical evidence in the academic literature has highlighted the critical role that socio-emotional skills play in cyberbullying perpetration and cyber victimization (57–60). Positive social and emotional development is pertinent as this critical part of the human developmental process influences a child’s self-confidence, empathy, ability to develop meaningful and lasting friendships and partnerships, and a sense of importance and value to those around the child. When children reach adolescence, changes in their brains, emotions, and bodies prime them to take on more complex social roles. A healthy socio-emotional development help adolescents have deeper conversations and better express and manage their emotions, whereas poor development in this domain will result in vice versa. Hence, developers of cyberbullying interventions should focus on theory-driven research designs, especially ones informed by the socio-emotional theory, before interventions are put into operational use.

It is also essential for future intervention-based researchers to appreciate the methodological strengths and limitations of systematic review and meta-analysis in planning, implementing, and evaluating high-quality research. This design of well-conducted, high-quality RCTs is the most robust method of synthesizing available data and is thus regarded as having the highest level of evidence (61). Finding the researched interventions that have, on average, made the most significant difference in effect size can be made with the help of systematic review and meta-analysis, both of which can provide essential insights into the current state of knowledge. Many practical guides are available that outline how to systematically and objectively conduct a meta-analysis in intervention research (62, 63). Although many challenges are associated with this design, including time-consuming screening and a thorough understanding of statistics, this methodology is more valuable than any single study contributing to the analysis because it can address the study size limitations, include a variety of populations, and allow for the evaluation of new hypotheses (64). Additionally, this research design allows for the integration of all evidence and the development of a coherent picture of the interventions’ effectiveness across theories, contexts, topics, ages, and intervention approaches (65). Hence, future intervention developers, practitioners, and policymakers should use a systematic review and meta-analysis to help them decide whether or not the intervention in question is empirically effective.

We cannot address cyberbullying by targeting only the subjects alone and expecting interventions to cause behavioral changes. Interventions must be designed and implemented systemically to address the challenge that cyberbullying poses. Hence, ensuring the engagement of all stakeholders, particularly field-level practitioners, is critical in identifying, prioritizing, and planning measures for intervention effectiveness. It is crucial to emphasize that addressing this issue is not just the responsibility of schools. Families, those who engage with young people, and the wider society must all have a role in preventing and reducing the harm caused by all types of bullying, including those that occur outside school hours. Strategies designed with a single-focus treatment, such as peer support programs (45), fail to address the problem holistically. Therefore, future studies must design interventions that involve all relevant stakeholders, including subjects, parents, policymakers, schools, and communities seeking participation and boosting their motivation, ability, and self-efficacy to prevent cyberbullying and management as a part of a holistic strategy that addresses cyberbullying as a shared responsibility.

### 4.2. Limitation

One of the significant limitations of this study is that not all eligible studies meet the rigorous criteria for inclusion in the meta-analysis. For example, although the study by Peng et al. (66) was rated as “high quality” based on the EPHPP checklist, further reviews revealed that the outcome of this study was presented as dichotomous data rather than continuous data (as used by the other four studies included in the meta-analysis), making analysis impossible. This limited number of studies prevented us from further determining the effect sizes of these interventions by doing subgroup analyses on gender, study designs, theoreticality, and intervention locations and broadening the age range literature search beyond non-school contexts. We could not conduct meta-regression analyses to estimate the effect of these covariates on cyberbullying behavior since the number of studies investigating these subgroups was small.

## 5. Conclusion

There is a critical need to establish all the previous recommendations with the most effective intervention design and structure to improve cyberbullying behaviors. Moving forward, researchers and practitioners in future studies should focus their educational efforts and investments better on interventions with theoretical grounding that can be implemented systemically. To combat cyberbullying effectively, researchers should devise theory-driven interventions, especially those based on socioemotional theories. The Collaborative for Academic, Social, and Emotional Learning (CASEL) framework highlights one such example of a systemic approach to implementing socio-emotional skills into students’ overall educational experiences that could lead to improved outcomes of decreasing bullying instances. Instead of limiting interventions to a single lesson or activity, socio-emotional learning (SEL) is integrated across key settings where students live and learn: classrooms, schools, homes, and communities. A systemic approach also ensures that school district and state policies, resources, and actions align together to support SEL. National policies at the macro level fundamentally play a role in creating ripe conditions for supportive environments and rich learning experiences. In essence, more high-quality research is required to identify the most effective cyberbullying interventions for youth by holistically involving all essential stakeholders.

## Data availability statement

The data utilized in this review are derived from previously published studies and are available upon request from the corresponding author.

## Author contributions

AMM contributed to the study’s conception and design. IK developed the search strategy, performed literature searches, quality assessments, and data extraction. AM conducted the statistical analyses, validation, and interpretation of data. AMM and IK contributed to manuscript preparation. All authors reviewed the manuscript and approved the submitted version.
