# Corrigendum: A systematic review and meta-analysis of interventions to decrease cyberbullying perpetration and victimization: an in-depth analysis within the Asia Pacific region

**DOI:** 10.3389/fpsyt.2023.1226698

**Published:** 2023-07-20

**Authors:** Ida Khairina Kamaruddin, Aini Marina Ma'rof, Ahmad Iqmer Nashriq Mohd Nazan, Habibah Ab Jalil

**Affiliations:** ^1^Faculty of Educational Studies, Universiti Putra Malaysia, Serdang, Malaysia; ^2^Faculty of Medicine and Health Sciences, Universiti Putra Malaysia, Serdang, Malaysia

**Keywords:** cyberbullying perpetration, cyberbullying victimization, intervention, systematic review and meta-analysis, Asia-Pacific

In the published article, there was an error in the **Data availability statement**. The error in the data availability statement in the original article is that PROSPERO determined that this review does not meet the eligibility criteria for inclusion in their database which is limited to systematic reviews with at least one direct health outcome. The correct **Data availability statement** appears below.

## Data availability statement

The data utilized in this review are derived from previously published studies and are available upon request from the corresponding author.

In the published article, some errors were made where the percentage of teens accessing online content from a mobile device was inaccurately quoted, and overlapping texts with other articles were erroneously included. We would like to rectify these errors by providing the following correction:

A correction has been made to **1. Introduction**, the first paragraph. This sentence previously stated:

“Before the pandemic caused by COVID-19, survey research indicated that 73% of teens aged 13–17 had smartphones and 91% reported accessing online content from a mobile device (1). Given the access to information and communication technology, it is not surprising that in the same survey, four out of five teens reported using the Internet “almost constantly” or “several times a day.” Throughout the pandemic, and once the pandemic subsides, youth and teens will continue to use technology regularly for school, extracurricular activities, and to engage with friends (2). One of the unfortunate consequences of the pervasive and prolonged use of technology is the cyberbullying phenomenon. Cyberbullying perpetration is the act of inflicting or receiving negative, damaging, or abusive language or harassment through information and communications technology (3). Over the past decade, prevalence rates for cyberbullying involvement among youth between the ages of 10 and 17 years (as a victim, bully, or bully-victim) have been reported to be between 14 and 21% (4–6). Meta-analytic findings revealed that approximately 15% of US students reported being victims or perpetrators of cyberbullying in the past 30 days (7). Prevalence rates vary widely in other countries, from a low 5.0% in Australia to a high 23.8% in Canada (8). A recent small-scale survey further suggests cyberbullying perpetration and victimization may have increased following the pandemic, perhaps due to students' increased technology use (9).”

The corrected sentence appears below:

“Surveys have revealed that 93% of teenagers owned smartphones and mobile devices were used by more than 90% of them to access online information before the COVID-19 outbreak (1). Technology will remain an essential part of their lives throughout the pandemic as well as after it (2). This heavy reliance on technology has resulted in an increase in cyberbullying. Cyberbullying perpetration is the act of sending, posting, or sharing negative, harmful, false, or mean content about someone else through various forms of digital technology (3). Cyberbullying has affected ~14–21% of youths over the past decade (either as a victim, a bully, or a bully-victim) (4–6). According to research conducted in the US, ~15% of students have experienced or perpetrated cyberbullying in the past 30 days (7). In other countries varying prevalence rates were reported, such as Australia at 5.0% and Canada at 23.8% (8). It appears that cyberbullying increased during the epidemic, perhaps due to students' intensive use of technology (9).”

In the published article, some errors were made where the overlapping texts with other articles were erroneously included. We would like to rectify these errors by providing the following correction:

A correction has been made to **1. Introduction**, “*1.2. Objectives*,” the first and second paragraphs. This sub-section was previously stated:

“This study aimed to conduct a comprehensive systematic review and meta-analysis of studies that measured the impacts of school violence, bullying, and targeted cyberbullying prevention programming on cyberbullying perpetration and victimization outcomes. Researchers have increased the implementation of interventions to target cyberbullying, and the results have been varied. We believe, despite several extensive systematic reviews and meta-analyses (for example, (2, 34, 35), that resynthesizing the various primary research findings is paramount in providing an appropriate, specific, and concrete response to cyber violence in policy and practice, particularly in the Asia Pacific region. For this project, we built upon credible previous meta-analytic work to conduct an updated systematic review and meta-analysis using comprehensive literature searches, thorough coding practices, and state-of-the-art meta-analysis techniques.

To address the research gap within the Asia-Pacific region on online user rights and protection concerning cyberbullying victims and perpetrators, this study aimed to provide further valuable empirical evidence by extending the work of the most recent large-scale systematic review and meta-analysis study on interventions to decrease cyberbullying perpetration and victimization (2) by expanding the age-range beyond school-aged settings.”

The corrected sentence appears below:

“This study seeks to comprehensively analyze studies examining interventions' effects on cyberbullying perpetration and victimization outcomes. Despite a number of extensive systematic reviews and meta-analyses [e.g., (2, 34, 35)], we consider it is vital to resynthesize the various primary research findings to provide a concrete and appropriate response to cyber violence in policy and practice, particularly in Asia Pacific region. To address the research gap within the Asia-Pacific region on online user rights and protection concerning cyberbullying victims and perpetrators, this study aimed to provide further valuable empirical evidence by extending the work of the most recent large-scale systematic review and meta-analysis study on interventions to decrease cyberbullying perpetration and victimization (2, 36) by expanding the age-range beyond school-aged settings.”

In the published article, we made an error regarding overlapping texts with other articles. The error we made was to directly reproduce the methods section from Polanin et al. (2021) without proper attribution under the wrongful assumption that replicating methods in scientific studies based on existing research is acceptable.

A correction has been made to the entire section **2. Methods**, “*2.1. Data Collection.”* This sub-section previously stated:

“**Intervention Studies:** Eligible studies must have tested the effects of an intervention to decrease cyberbullying perpetration and victimization. Studies were not excluded based on the type of intervention tested; that is, a wide range of interventions and programs were included, which provides a robust database of studies. Studies on direct interventions were included in which study authors implemented cyberbullying intervention programs specifically intended to reduce cyberbullying perpetration and victimization. We also included interventions such as general violence prevention programs, physical aggression, and bullying prevention programs, and school climate models.

**Comparison Group:** The study must have included an eligible comparison group to be included in the review. Several eligible comparison groups may have been used, such as those that received no intervention, treatment as usual, or minimal or proven-to-be ineffective treatment. For the comparison group to be eligible, the study must have demonstrated that the minimal treatment was ineffective.

**Research Design:** We included studies that randomly assigned participants to a condition (randomized controlled trials) and studies that non-randomly assigned participants (quasi-experimental designs). In light of the number of studies that assign classrooms and schools to conditions, we did not exclude any studies based on the level of assignment. Hence, we included studies that may have randomly or non-randomly assigned classrooms, schools, or school districts to conditions.

**Primary Outcome Measures:** If primary studies did not implement a direct cyberbullying intervention, they had to have measured a cyberbullying perpetration or victimization outcome variable to be included in the review. If the authors implemented a general violence or bullying prevention program but did not include a cyberbullying measure, we did not immediately exclude it. This procedure and the reasoning behind it have been explained by Polanin et al. (37). They found that excluding the identified studies would change some substantive conclusions in their meta-analysis. Another rationale behind this is that recent meta-analytic research indicated that traditional in-person bullying perpetration and victimization and cyberbullying perpetration and victimization are correlated (26).

**Timeframe:** We expected that the vast majority of studies would have been published on or after 2003 because that was the earliest date for consistent mentioning of the terms electronic bullying, computer bullying, and cyberbullying in the literature. To ensure we synthesized all studies, we included any studies published on or after 1995.

**Publication Status:** We included all types of study reports, published or unpublished, to ensure that every available study report would be included in the review and decreased the well-known upward bias of studies published in peer-reviewed journals (38). We comprehensively searched for and attempted to locate all unpublished datasets that included cyberbullying perpetration and victimization measures.

**Language and Country of Origin:** Studies must have been published in English or Bahasa Melayu, which represented the native languages of our team members. We did not exclude studies based on country of origin (i.e., where a study's sample originated).”

The corrected sentence appears below:

“**Intervention Studies**. Studies on interventions designed to reduce cyberbullying perpetration and victimization were included in this review, regardless of the type of intervention. This gave us a wide range of studies to draw from, including those focusing on direct interventions, as well as those exploring broader violence prevention initiatives and anti-bullying programs.

**Comparison Group**. For the study to be considered for the review, it was required to contain a comparison group that met specific eligibility criteria. The comparison group could have been composed of individuals who did not receive any form of intervention, those who underwent treatment as per usual practice, or those who received a treatment that was either minimal or demonstrated to be ineffective. The comparison group was necessary to provide a point of reference for evaluating the effectiveness of the intervention being studied. Without such a group, it would be difficult to draw any meaningful conclusions about the efficacy of the intervention in question.

**Research Design**. We included randomized controlled trials, quasi-experimental studies, and studies that may have assigned groups in a randomized or non-randomized way to conditions without any exclusion based on the level of assignment.

**Primary Outcome Measures**. This review included studies that assessed cyberbullying perpetration or victimization as the primary outcome measure and did not exclude those that utilized a broader program to prevent violence or bullying instead of a specific cyberbullying intervention. This procedure and the reasoning behind it have been explained by Polanin et al. (38). The exclusion of certain studies was found to alter significant conclusions in their meta-analysis. Another rationale for this is previous meta-analytic studies' finding that the perpetration and victimization of conventional bullying and cyberbullying are connected (26).

**Time range**. Although cyberbullying-related terms started appearing in literature around 2003, studies published since 1995 were also included to ensure comprehensive coverage of research.

**Publication Status**. To minimize publication bias, we searched for relevant information on cyberbullying, including published and unpublished research reports and available data sets (39) with cyberbullying perpetration and victimization measures.

**Language**. Publications must be in English or Bahasa Melayu, regardless of the country of origin, were included in our review.”

In the published article, we made an error regarding overlapping texts with other articles. The error we made was to directly reproduce the methods section from Polanin et al. (2021) without proper attribution under the wrongful assumption that replicating methods in scientific studies based on existing research is acceptable.

A correction has been made to **Methods**, “*2.2. Literature Search and Screening*.” This sub-section previously stated:

“We used several complementary approaches, including searches of the traditional and gray works of literature, forward and backward reference harvesting, and hand searching of targeted journals. First, we conducted an electronic bibliographic literature search to identify qualifying studies. We then searched the following online databases, which included both published and unpublished studies, using search terms tailored to each database available through our University's library services: Cambridge Journal Online, EBSCOHOST, ERIC, IEEE XPLORE, Oxford Journal Online, ProQuest Dissertations and Theses, PubMed (Medline), Science Direct, Scopus, and Springerlink.”

The corrected sentence appears below:

“We employed multiple methods to identify qualifying studies, including electronic bibliographic searches and forward and backward reference harvesting. Our search included published and unpublished works within the traditional and gray literature. We used tailored search terms for each database and the following online databases available through our University's library services: Cambridge Journal Online, EBSCOhost, ERIC, IEEE XPLORE, Oxford Journal Online, ProQuest Dissertations and Theses, PubMed (Medline), Science Direct, Scopus, and SpringerLink.”

In the published article, we made an error regarding overlapping texts with other articles. The error we made was to directly reproduce the methods section from Polanin et al. (2021) without proper attribution under the wrongful assumption that replicating methods in scientific studies based on existing research is acceptable.

A correction has been made to **2. Methods**, “*2.3. PRISMA Flowchart*.” This sub-section previously stated:

“**2.3.1. Abstract screening**

We used an exhaustive methodology to screen the large number of studies identified in this round (detailed in 2). We developed an abstract screening guide (see **Supplementary Data Sheet 2**) and screened the abstracts (Supplementary Table 1) using the free Rayyan software (39), which provides open-source web-based abstract screening. All review team members screened abstracts.

**2.3.2. Full-text retrieval**

Team members located full-text PDFs for all abstracts that were screened during the first round of screening in preparation for a second round using a full-text screening tool.

**2.3.3. Full-text screening**

We organized the results from all phases of the project (i.e., search results, abstract screening, full-text screening, and data extraction), and team members entered full-text screening responses into an “eligibility screen.” we ensured the accuracy of the screening process as all the “keep” or “drop” results were validated by the leading research members (i.e., the principal investigator or the lead statistician). As with abstract screening, team members conducted in-house training led by the lead statistician, after which we conducted the pilot screening.

**2.3.4. Data extraction**

A codebook detailed all information extracted from each study, and we further developed the relational database in Excel. We extracted study-level information such as details on the sample demographics and how the individuals were placed in groups, characteristics of the intervention and comparison conditions (including who developed and implemented the intervention and information on implementation fidelity), construct-level information (such as how the predictor and outcome variables were measured), and the summary data that could be used to estimate effect sizes (such as semi-partial correlations and/or adjusted-odds ratios derived from a regression model). Coders extracted information about each study and entered it into Excel coding screens dedicated to that information (e.g., samples, conditions, constructs, and effect sizes).

The corrected sentence appears below:

“**2.3.1. Abstract Screening**

We employed a comprehensive approach to review the numerous studies located during this round of research (as outlined in 2 and 36). All members of the review team assessed the abstracts. We developed a screening guide for abstracts (see **Supplementary material**) and utilized the free Rayyan software (40) for web-based abstract screening.

**2.3.2. Full-Article Retrieval**

To prepare for the next screening stage, the team members retrieved the complete article PDFs of the previously screened titles and abstracts.

**2.3.3. Full-Article Screening**

The team carried out a thorough screening process for eligibility by entering responses into a designated tool, followed by review and validation by the principal investigator and lead statistician, and after a training session, a pilot screening was conducted.

**2.3.4. Data Extraction**

We created a codebook to document all data that was extracted from each study. The data comprised demographics of the sample, characteristics of the intervention and comparison conditions, and summary statistics useful for effect size estimation. An Excel-based relational database was designed to structure the information. To maintain accuracy and consistency, coders used dedicated coding screens in Excel for each category of extracted data.”

In the published article, we made an error regarding overlapping texts with other articles. The error we made was to directly reproduce the methods section from Polanin et al. (2021) without proper attribution under the wrongful assumption that replicating methods in scientific studies based on existing research is acceptable.

A correction has been made to **Methods**, “*2.4. Data Analysis*.” This sub-section previously stated:

“We conducted separate analyses for each outcome variable category: (1) cyberbullying perpetration and (2) cyberbullying victimization. We reported summary statistics for the included studies, e.g., publication status, program target, research design, and location. We also planned to perform a sub-analysis looking further into the potential differentiated effects of gender, randomized controlled trial versus non-randomized control trial designs, whether or not the studies were theory-based or non-theory-based, and geographical locations with a specific focus on Asia-Pacific regions, and studies that also covers the age-range beyond K-12.

**2.4.1. Meta-analyses**

First, we estimated separate meta-analytic models that predict the two primary outcome variable categories. We used a random-effects model with robust variance estimation (40), which weights each effect size by the inverse of its variance (41) to produce a weighted average of the effect sizes.

Next, we planned to conduct two confirmatory meta-regression analyses predicting each behavioral outcome variable (i.e., cyberbullying perpetration and cyberbullying victimization). The meta-regression could be conducted using the following predictor variables: (1) country of origin (i.e., Asia-Pacific versus non-Asia Pacific); (2) program target (i.e., specifically targeted cyberbullying versus did not specifically target cyberbullying); (3) timepoint of second measurement (i.e., posttest versus follow up); (4) effect size type (i.e., dichotomous versus continuous), (5) percentage of males, and (6) the percentage of ethnic minority participants. Sub-analyses would be conducted to evaluate the potential moderating effects of gender, comparing randomized controlled trial study designs versus nonrandomized control trial designs, whether or not the studies were theory-based or non-theory-based, taking geographical locations of subjects into consideration, and expanding the age range beyond school-aged settings.

**2.4.2. Exploratory analysis**

Finally, we analyzed the overall effect sizes for each of the named programs identified through our systematic review.”

The corrected sentence appears below:

“We conducted separate analyses for each outcome variable category: (1) cyberbullying perpetration and (2) cyberbullying victimization. The characteristics of the studies included in the analysis were documented, including the publication status, the target of the program, the type of research design, and geographical location. We also planned to perform a sub-analysis looking further into the potential differentiated effects of gender, randomized controlled trial vs. non-randomized control trial designs, whether or not the studies were theory-based or non-theory-based, and geographical locations with a specific focus on Asia-Pacific regions, and studies that also covers the age-range beyond K-12.

**2.4.1. Meta-Analyses**

We analyzed two primary outcome variables using meta-analytic models, with separate analyses using a random-effects model and robust variance estimation method (41). The random-effects model considered both within-study and between-study variations, making it suitable for studies with diverse populations and designs. The robust variance estimation method was used to estimate standard errors of effect size estimates and to adjust for potential biases due to small sample sizes or heterogeneity. Each effect size estimate was weighted by its inverse variance to calculate the average effect size (42). The model assigned greater importance to effect sizes with smaller variances, resulting in a more accurate estimation of the overall effect size.

Our original plan, which followed the preceding step, involved conducting two confirmatory meta-regression analyses to investigate the potential predictors contributing to cyberbullying perpetration and victimization. Our meta-regression approach would involve incorporating several pertinent variables. These variables would encompass the type of effect size, the objectives of the intervention, the native country of the study participants, the timing of the follow-up measurement, as well as the percentage of male participants and individuals from ethnic minority groups. Sub-analyses were also planned to examine potential moderating effects of gender, study design, theory-based or non-theory-based, geographical location, and age range. Sub-analyses would be conducted to evaluate the potential moderating effects of gender, comparing randomized controlled trial study designs vs. non-randomized control trial designs, whether or not the studies were theory-based or non-theory-based, taking geographical locations of subjects into consideration, and expanding the age range beyond school-aged settings.

**2.4.2. Exploratory Analysis**

Ultimately, we performed an exploratory analysis aimed at assessing the overall effect size of the specified interventions that were identified during our review process.”

In the published article, several errors were made regarding the PROSPERO registration. We have deleted the below sentences from the **Abstract** and **Methods** sections, respectively:

“Systematic Review Registration: https://www.crd.york.ac.uk/prospero/, identifier: CRD42022313369.

The study was registered in the PROSPERO International Prospective Register of Systematic Reviews (registration number CRD42022313369), and the detailed prespecified protocol is available online.”

In the published article, there is an error in the reference list. The updated reference list incorporates a new reference entry identified as 36, which necessitates renumbering the preexisting reference labeled as 36 to be revised as 37. Consequently, all subsequent numerical references from 37 to 66 have been incremented by 1 in both the in-text citations and the reference list.

The authors apologize for the errors and state that this does not change the scientific conclusions of the article in any way. The original article has been updated.
