# Supplementary material for: A systematic review and meta-analysis of interventions to decrease cyberbullying perpetration and victimization: An in-depth analysis within the Asia Pacific region
Source: Front Psychiatry. 2023 Jan 27;14:1014258. doi: 10.3389/fpsyt.2023.1014258 (PMC9911532; doi:10.3389/fpsyt.2023.1014258)
Supplement: Supplementary file 1 [file Data_Sheet_1.PDF]

Document 1

Summary of Database Search

**Database**	**ProQ**	**Science Direct**				**Ebsco**	**Scopus**	**Oxford**	**Springer**			**ERIC**	**IEEE**	**PubMed**		**Cambr**	**TOTAL**
**Search #**	**1k**	**2h**	**2i**	**2j**	**2k**	**3i**	**4l**	**5c**	**6i**	**6j**	**6h**	**7h**	**8e**	**11j**	**11l**	**12f**	
**Results**	254	68	199	173	141	287	393	55	150	239	142	231	41	86	42	39	
**Total**	254	581				287	393	55	531			231	41	128		39	**2,540**
**Imported***	253	581				244	393	55	531			231	41	125		39	**2,493**

**After records were removed automatically the by database*

Databases:

1. ProQ: ProQuest Dissertations & Theses (PQDT) Global
2. Science Direct: Science Direct
3. Ebsco: EBSCOhost
4. Scopus: Scopus
5. Oxford: Oxford Journals Collection
6. Springer: SpringerLink
7. ERIC: ERIC (Education Resources Information Center)
8. IEEE: IEEE Xplore
9. PubMed: PubMed
10. Cambr: Cambridge Core
