# Supplementary material for: A systematic review and meta-analysis of interventions to decrease cyberbullying perpetration and victimization: An in-depth analysis within the Asia Pacific region
Source: Front Psychiatry. 2023 Jan 27;14:1014258. doi: 10.3389/fpsyt.2023.1014258 (PMC9911532; doi:10.3389/fpsyt.2023.1014258)
Supplement: Supplementary file 2 [file Data_Sheet_2.PDF]

Document 2

Abstract Screening Guide

Citation, Title, and Abstract Screening

1. Does the **citation** indicate publication on or after 1995?
    a. Yes: continue screening
    b. No: stop screening

2. Does the **title or abstract** use English or Malay?
    a. Yes: continue screening
    b. No: stop screening

3. Does the **title or abstract NOT** indicate that a cyberbullying systematic review or meta-analysis was conducted?
    a. Yes: continue screening
    b. No: stop screening

4. Does the **title or abstract** indicate that this is NOT a correction or erratum?
    a. Yes: continue screening
    b. No: stop screening

Abstract Screening

5. Does the **abstract** indicate that this study was longitudinal?
    a. Yes or Unsure/Unclear: continue screening

        - Key words: prospective, over time, trajectory, panel, waves, multiple time points, time 1, time 2, T1, T2, school transition

    b. No: stop screening

        -For example: the study only used cross-sectional, prevalence, rate, incidence, or all data collected at the same time

        -Key point: studies that say they used “only wave 3” indicate that the abstract is cross-sectional

6. Does the **abstract** indicate that cyberbullying was studied?
    a. Yes or Unsure/Unclear: continue screening

       -Key words: "electronic bull*" OR "internet bull*" OR "cyber abuse" OR "cyber harass*" OR “cyber-harass*” OR cyberharass* OR cyberthreat* OR "cyber threat*" OR "cyber-threat*" OR cyberbull* OR "cyber bull*" OR "cyber-bull*" OR cyberstalk* OR "cyber stalk*" OR "cyber-stalk*" OR cyberaggress* OR "cyber aggress*" OR cyber-aggress* OR "cyber victim*" OR cyber-victim* OR cybervictim* OR "social media" OR "instant messag*" OR "electronic communication" OR Sextortion OR “online bull*” OR "bystander cyberbull*" OR "cyber mobbing" OR cybermobbing OR "cyberbullying perpetration" OR "cyber violence" OR "digital bullying" OR "E-bullying" OR "online victimization" OR "online violence" OR "online harassment" OR "online aggression" OR "phone bullying" OR "SMS bullying" OR "text bullying" OR "virtual aggression" OR "virtual mobbing" OR "internet harassment" OR "internet victimization" OR "internet aggression"

    b. No: stop screening

       -Other constructs, in the absence of cyberbully measures above, **not** eligible: suicide, PTSD, “risk factors”, “high-risk behaviors”, substance use or abuse, sibling violence, antisocial behaviors (not specifically mentioning bullying or violence), conduct disorder, disruptive behavior, misbehavior, problem behaviors

7. Does the **abstract** indicate that the study uses a quantitative design?
    a. Yes or Unsure/Unclear: continue screening

       -Key words: regression, covariate, modeling, structural equation modeling, mean, standard deviation, correlation, variance, causal, experiment, QED, randomized controlled trial (RCT), propensity score matching, Quasi-experimental

    b. No: stop screening

       -For example: qualitative only: ethnography, action research, social observation, focus groups, case study research

Decision: **Should this article be included?**

a. **Yes**, all 7 screening questions answered Yes or Unclear

b. **No**, at least one answers definitely “No”

This guideline was prepared based on:

Polanin, J. R., Pigott, T. D., Espelage, D. L., & Grotpeter, J. K. (2019). Best practice guidelines for abstract screening large-evidence systematic reviews and meta-analyses. *Research Synthesis Methods, 10*(3), 330–342. https://doi.org/10.1002/jrsm.1354
