# Supplementary material for: A systematic review and meta-analysis of interventions to decrease cyberbullying perpetration and victimization: An in-depth analysis within the Asia Pacific region
Source: Front Psychiatry. 2023 Jan 27;14:1014258. doi: 10.3389/fpsyt.2023.1014258 (PMC9911532; doi:10.3389/fpsyt.2023.1014258)
Supplement: Supplementary file 3 [file Data_Sheet_3.PDF]

Document 3

Keyterms

Keyterm 1	intervention* OR evaluat* OR impact* OR effectiveness OR prevention OR program OR effect OR efficacy
Keyterm 2	causal OR "control group*" OR "comparison group*" OR "matched group*" OR random* OR treatment OR experiment OR QED* OR RCT OR "propensity score matching" OR quasi-experimental
Keyterm 3	"electronic bull*" OR "internet bull*" OR "cyber abuse" OR "cyber harass*" OR "cyber-harass*" OR cyberharass* OR cyberthreat* OR "cyber threat*" OR "cyber-threat*" OR cyberbull* OR "cyber bull*" OR "cyber-bull*" OR cyberstalk* OR "cyber stalk*" OR "cyber-stalk*" OR cyberaggress* OR "cyber aggress*" OR cyber-aggress* OR "cyber victim*" OR cyber-victim* OR cybervictim* OR "social media" OR "instant messag*" OR "electronic communication" OR Sextortion OR "online bull*" OR "bystander cyberbull*" OR "cyber mobbing" OR cybermobbing OR "cyberbullying perpetration" OR "cyber violence*" OR "digital bullying" OR "E-bullying" OR "online victimi?ation" OR "online violence*" OR "online harassment" OR "online aggression" OR "phone bullying" OR "SMS bullying" OR "text bullying" OR "virtual aggression" OR "virtual mobbing" OR "internet harassment" OR "internet victimi?ation" OR "internet aggression" OR "online abuse"
Keyterm 4	"Asia-Pacific" OR "South-asia" OR "East-asia" OR "Southeast asia" OR "South-east asia" OR "north-asia" OR oceania OR Australia OR Brunei OR Myanmar OR Burma OR Cambodia OR China OR Fiji OR Indonesia OR Japan OR Kiribati OR Laos OR Malaysia OR "Marshall Islands" OR Micronesia OR Mongolia OR Nauru OR "New Zealand" OR "North Korea" OR Palau OR "Papua New guinea" OR Philippines OR Samoa OR Singapore OR "Solomon Islands" OR "South Korea" OR Taiwan OR Thailand OR "Timor-leste" OR Tonga OR Tuvalu OR Vanuatu OR Vietnam

Note: These search terms were developed based on a combination of the most recent high-quality meta-analysis of cyberbullying intervention studies, as follows:

1. Polanin, J. R., Espelage, D. L., Grotpeter, J. K., Ingram, K., Michaelson, L., Spinney, E., Valido, A., Sheikh, A. E., Torgal, C., & Robinson, L. (2021). A Systematic Review and Meta-analysis of Interventions to Decrease Cyberbullying Perpetration and Victimization. *Prevention Science*. https://doi.org/10.1007/s11121-021-01259-y
2. Evangelio, C., Rodríguez-González, P., Fernández-Río, J., & Gonzalez-Villora, S. (2022). Cyberbullying in elementary and middle school students: A systematic review. *Computers & Education*, *176*. https://doi.org/10.1016/j.compedu.2021.104356
3. Lan, M., Law, N., & Pan, Q. (2022). Effectiveness of anti-cyberbullying educational programs: A socio-ecologically grounded systematic review and meta-analysis. *Computers in Human Behavior*, *130*. https://doi.org/10.1016/j.chb.2022.107200
