# Supplementary material for: A systematic review and meta-analysis of interventions to decrease cyberbullying perpetration and victimization: An in-depth analysis within the Asia Pacific region
Source: Front Psychiatry. 2023 Jan 27;14:1014258. doi: 10.3389/fpsyt.2023.1014258 (PMC9911532; doi:10.3389/fpsyt.2023.1014258)
Supplement: Supplementary file 4 [file Data_Sheet_4.PDF]

Document 4

Search Terms and Strings

#	Date	Database	Search Terms	Results	Notes
1k	25/2/2022	ProQuest	mainsubject.Exact("cyberbullying") AND AB,TI(intervention* OR evaluat* OR impact* OR effectiveness OR prevention OR program OR effect OR efficacy) AND FT((causal OR "control group*" OR "comparison group*" OR "matched group*" OR random* OR treatment OR experiment OR QED* OR RCT OR "propensity score matching" OR Quasi-experimental) AND ("Asia-Pacific" OR "South-asia" OR "East-asia" OR "Southeast asia" OR "South-east asia" OR "north-asia" OR oceania OR Australia OR Brunei OR Myanmar OR Burma OR Cambodia OR China OR Fiji OR Indonesia OR Japan OR Kiribati OR Laos OR Malaysia OR "Marshall Islands" OR Micronesia OR Mongolia OR Nauru OR "New Zealand" OR "North Korea" OR Palau OR "Papua New guinea" OR Philippines OR Samoa OR Singapore OR "Solomon Islands" OR "South Korea" OR Taiwan OR Thailand OR "Timor-leste" OR Tonga OR Tuvalu OR Vanuatu OR Vietnam))	254	• Command Line Search in Advanced Search<br>• results outside my library's subscription<br>• Jan 1 1995 - Feb 22 2022<br>• Exclude duplicate documents<br>• Applied filter: English
2h	16/2/2022	Science Direct	**Title, abstract or author-specified keywords**<br>“online abuse” OR "online bully" OR "electronic bully" OR "internet bully" OR cyberabuse OR "cyber abuse" OR "cyber harass"	68	
2i			**Title, abstract or author-specified keywords**<br>"cyber bully" OR cyberstalk OR "cyber stalk" OR Sextortion OR "digital bullying" OR "phone bullying" OR "text bullying"	199	
2j			**Title, abstract or author-specified keywords**<br>"cyberbullying perpetration" OR "cyber violence" OR "E-bullying" OR "online victimi?ation" OR "online violence" OR "online harassment" OR "online aggression" OR "internet harassment" OR "cyber mobbing"	173	
2k			**Title, abstract or author-specified keywords**<br>cyberbully OR cybermobbing OR "SMS bullying" OR "virtual aggression" OR "virtual mobbing" OR "internet victimi?ation" OR "internet aggression" OR cybervictim OR "cyber victim"	141	

3i	12/2/ 2022	EBSCOHost	**(SU Subject Terms)**<br>"electronic bull*" OR "internet bull*" OR "cyber abuse" OR "cyber harass*" OR "cyber-harass*" OR cyberharass* OR cyberthreat* OR "cyber threat*" OR "cyber-threat*" OR cyberbull* OR "cyber bull*" OR "cyber-bull*" OR cyberstalk* OR "cyber stalk*" OR "cyber-stalk*" OR cyberaggress* OR "cyber aggress*" OR cyber-aggress* OR "cyber victim*" OR cyber-victim* OR cybervictim* OR Sextortion OR "online bull*" OR "bystander cyberbull*" OR "cyber mobbing" OR cybermobbing OR "cyberbullying perpetration" OR "cyber violence*" OR "digital bullying" OR "E-bullying" OR "online victimi?ation" OR "online violence*" OR "online harassment" OR "online aggression" OR "phone bullying" OR "SMS bullying" OR "text bullying" OR "virtual aggression" OR "virtual mobbing" OR "internet harassment" OR "internet victimi?ation" OR "internet aggression" OR "online abuse"<br><br>**AND (AB Abstract)**<br>intervention* OR evaluat* OR impact* OR effectiveness OR prevention OR program OR effect OR efficacy<br><br>**AND (TX All Text)**<br>Causal OR "control group*" OR "comparison group*" OR "matched group*" OR random* OR treatment OR experiment OR QED* OR RCT OR "propensity score matching" OR Quasi-experimental<br><br>**AND (TX All Text)**<br>"Asia-Pacific" OR "South-asia" OR "East-asia" OR "Southeast asia" OR "South-east asia" OR "north-asia" OR oceania OR Australia OR Brunei OR Myanmar OR Burma OR Cambodia OR China OR Fiji OR Indonesia OR Japan OR Kiribati OR Laos OR Malaysia OR "Marshall Islands" OR Micronesia OR Mongolia OR Nauru OR "New Zealand" OR "North Korea" OR Palau OR "Papua New guinea" OR Philippines OR Samoa OR Singapore OR "Solomon Islands" OR "South Korea" OR Taiwan OR Thailand OR "Timor-leste" OR Tonga OR Tuvalu OR Vanuatu OR Vietnam	287	• Search in:<br>1) Academic Search Complete,<br>2) MEDLINE, and<br>3) Psychology and Behavioral Sciences Collection<br>• Apply related words<br>• Also search within the full text of the articles<br>• Apply equivalent subjects<br>• Note: Exact duplicates removed from the results.<br>• Publication Date: 2008-2022
4l	23/2/ 2021	SCOPUS	**TITLE-ABS-KEY**(("electronic bull*" OR "internet bull*" OR "cyber abuse" OR "cyber harass*" OR "cyber-harass*" OR cyberharass* OR cyberthreat* OR "cyber threat*" OR "cyber-threat*" OR cyberbull* OR "cyber bull*" OR "cyber-bull*" OR cyberstalk* OR "cyber stalk*" OR "cyber-stalk*" OR cyberaggress* OR "cyber aggress*" OR cyber-aggress* OR "cyber victim*" OR cyber-victim* OR cybervictim* OR Sextortion OR "online bull*" OR "bystander cyberbull*" OR "cyber mobbing" OR cybermobbing OR "cyberbullying perpetration" OR "cyber violence*" OR "digital bullying" OR "E-bullying" OR "online victimization" OR "online violence*" OR "online harassment" OR "online aggression" OR "phone bullying" OR	393	

			"SMS bullying" OR "text bullying" OR "virtual aggression" OR "virtual mobbing" OR "internet harassment" OR "internet victimization" OR "internet aggression" OR "online abuse") **AND** (intervention* OR evaluat* OR impact* OR effectiveness OR prevention OR program OR effect OR efficacy) AND (Causal OR "control group*" OR "comparison group*" OR "matched group*" OR random* OR treatment OR experiment OR QED* OR RCT OR "propensity score matching" OR Quasi-experimental) **AND NOT** (bulletin) AND NOT cancer) **AND ALL**("Asia-Pacific" OR "South-asia" OR "East-asia" OR "Southeast asia" OR "South-east asia" OR "north-asia" OR oceania OR Australia OR Brunei OR Myanmar OR Burma OR Cambodia OR China OR Fiji OR Indonesia OR Japan OR Kiribati OR Laos OR Malaysia OR "Marshall Islands" OR Micronesia OR Mongolia OR Nauru OR "New Zealand" OR "North Korea" OR Palau OR "Papua New guinea" OR Philippines OR Samoa OR Singapore OR "Solomon Islands" OR "South Korea" OR Taiwan OR Thailand OR "Timor-leste" OR Tonga OR Tuvalu OR Vanuatu OR Vietnam) **AND PUBYEAR** > 1994 **AND** (LIMIT-TO (LANGUAGE,"English"))		
5c	26/2/ 2021	Oxford	**Abstract:**<br>"electronic bull*" OR "internet bull*" OR "cyber abuse" OR "cyber harass*" OR “cyber-harass*” OR cyberharass* OR cyberthreat* OR "cyber threat*" OR "cyber-threat*" OR cyberbull* OR "cyber bull*" OR "cyber-bull*" OR cyberstalk* OR "cyber stalk*" OR "cyber-stalk*" OR cyberaggress* OR "cyber aggress*" OR cyber-aggress* OR "cyber victim*" OR cyber-victim* OR cybervictim* OR Sextortion OR “online bull*” OR "bystander cyberbull*" OR "cyber mobbing" OR cybermobbing OR "cyberbullying perpetration" OR "cyber violence*" OR "digital bullying" OR "E-bullying" OR "online victimi?ation" OR "online violence*" OR "online harassment" OR "online aggression" OR "phone bullying" OR "SMS bullying" OR "text bullying" OR "virtual aggression" OR "virtual mobbing" OR "internet harassment" OR "internet victimi?ation" OR "internet aggression" OR “online abuse”<br>**Full Text:**<br>Australia OR Brunei OR Burma OR Myanmar OR Cambodia OR China OR Fiji OR Indonesia OR Japan OR Kiribati OR Laos OR Malaysia OR “Marshall Islands” OR Micronesia OR Mongolia OR Nauru OR “New Zealand” OR “North Korea” OR Palau OR “Papua New Guinea” OR Philippines OR Samoa OR Singapore OR “Solomon Islands” OR “South Korea” OR Taiwan OR Thailand OR “Timor-Leste” OR Tonga OR Tuvalu OR Vanuatu OR Vietnam	55	**Published:** January 1995 to February 2022
6i	25/2/ 2022	Springerlink	**with all of the words**<br>perpetrat* victim*<br><br>**Exact phrases:**	150	**Within:** 1995-2022<br>**Language: English**<br><br>**Content type:** Article

			quasi experimental<br><br>**With at least one of the words:**<br>"electronic bull*" "internet bull*" "cyber abuse" "cyber harass*" "cyber-harass*" cyberharass* cyberthreat* "cyber threat*" "cyber-threat*" cyberbull* "cyber bull*" "cyber-bull*" cyberstalk* "cyber stalk*" "cyber-stalk*" cyberaggress* "cyber aggress*" cyber-aggress* "cyber victim*" cyber-victim Sextortion "online bull*" "bystander cyberbull*" "cyber mobbing" cybermobbing "cyberbullying perpetration" "cyber violence*" "digital bullying" "E-bullying" "online victimi?ation" "online violence*" "online harassment" "online aggression" "phone bullying" "SMS bullying" "text bullying" "virtual aggression" "virtual mobbing" "internet harassment" "internet victimi?ation" "internet aggression" "online abuse"<br><br>**Without the words**<br>Systematic review		Include Preview-Only content
6j			**with all of the words**<br>perpetration victimization<br><br>**Exact phrases:**<br>Randomized controlled trial<br><br>**With at least one of the words:**<br>"electronic bull*" "internet bull*" "cyber abuse" "cyber harass*" "cyber-harass*" cyberharass* cyberthreat* "cyber threat*" "cyber-threat*" cyberbull* "cyber bull*" "cyber-bull*" cyberstalk* "cyber stalk*" "cyber-stalk*" cyberaggress* "cyber aggress*" cyber-aggress* "cyber victim*" cyber-victim Sextortion "online bull*" "bystander cyberbull*" "cyber mobbing" cybermobbing "cyberbullying perpetration" "cyber violence*" "digital bullying" "E-bullying" "online victimization" "online violence*" "online harassment" "online aggression" "phone bullying" "SMS bullying" "text bullying" "virtual aggression" "virtual mobbing" "internet harassment" "internet victimization" "internet aggression" "online abuse"<br><br>**Without the words**<br>Systematic review	239	
6h			**With at least one of the words:**<br>intervention* evaluat* impact* effectiveness prevention program effect efficacy<br><br>**where the title contains**	142	

			cyberbullying<br><br>**Without the words**<br>detection		
7g	24/2/<br>2022	ERIC	**AB,TI**(("electronic bull*" OR "internet bull*" OR "cyber abuse" OR "cyber harass*" OR "cyber-harass*" OR cyberharass* OR cyberthreat* OR "cyber threat*" OR "cyber-threat*" OR cyberbull* OR "cyber bull*" OR "cyber-bull*" OR cyberstalk* OR "cyber stalk*" OR "cyber-stalk*" OR cyberaggress* OR "cyber aggress*" OR cyber-aggress* OR "cyber victim*" OR cyber-victim* OR cybervictim* OR "social media" OR "instant messag*" OR "electronic communication" OR Sextortion OR "online bull*" OR "bystander cyberbull*" OR "cyber mobbing" OR cybermobbing OR "cyberbullying perpetration" OR "cyber violence*" OR "digital bullying" OR "E-bullying" OR "online victimization" OR "online violence*" OR "online harassment" OR "online aggression" OR "phone bullying" OR "SMS bullying" OR "text bullying" OR "virtual aggression" OR "virtual mobbing" OR "internet harassment" OR "internet victimization" OR "internet aggression" OR "online abuse") **AND** (intervention* OR evaluat* OR impact* OR effectiveness OR prevention OR program OR effect OR efficacy)) **AND** ("Asia-Pacific" OR "South-asia" OR "East-asia" OR "Southeast asia" OR "South-east asia" OR "north-asia" OR oceania OR Australia OR Brunei OR Myanmar OR Burma OR Cambodia OR China OR Fiji OR Indonesia OR Japan OR Kiribati OR Laos OR Malaysia OR "Marshall Islands" OR Micronesia OR Mongolia OR Nauru OR "New Zealand" OR "North Korea" OR Palau OR "Papua New guinea" OR Philippines OR Samoa OR Singapore OR "Solomon Islands" OR "South Korea" OR Taiwan OR Thailand OR "Timor-leste" OR Tonga OR Tuvalu OR Vanuatu OR Vietnam)	231	• Exclude duplicate documents<br>• Include: Spelling variants for your search terms<br>• Additional limits - Date: From January 01 1995 to February 16 2022<br>• Applied filters: Scholarly Journals
8e	27/2/<br>2022	IEEE Xplore	**(ABSTRACT)**<br>"cyber abuse" OR cyber-harass* OR cyberthreat* OR cyber-threat* OR cyberbull* OR cyber-bull* OR cyber-aggress* OR Sextortion OR “online bull*” OR "cyberbullying perpetration" OR "cyber violence" OR "digital bullying" OR "E-bullying" OR "online victimi?ation" OR "online violence" OR "online harassment" OR "online aggression" OR "phone bullying" OR "SMS bullying" OR "text bullying" OR "virtual aggression" OR "virtual mobbing" OR "internet harassment" OR "internet victimi?ation" OR "internet aggression" OR “cyber-victim” OR “cyber-stalk” OR cyberstalk<br><br>**AND (ABSTRACT)**<br>intervention* OR evaluat* OR impact* OR effectiveness OR Prevention OR	41	• **Filters Applied:** 1995 - 2022

			program OR effect OR efficacy<br><br>**AND (ALL METADATA)**<br>Causal OR "control group*" OR "comparison group*" OR "matched group*" OR random* OR treatment OR experiment OR QED* OR RCT OR "propensity score matching" OR Quasi-experimental<br><br>**AND (ALL METADATA)**<br>Australia OR Brunei OR Myanmar OR Cambodia OR China OR Fiji OR Indonesia OR Japan OR Kiribati OR Laos OR Malaysia OR "Marshall Islands" OR Micronesia OR Mongolia OR Nauru OR "New Zealand" OR "North Korea" OR Palau OR "Papua New Guinea" OR Philippines OR Samoa OR Singapore OR "Solomon Islands" OR "South Korea" OR Taiwan OR Thailand OR "Timor-Leste" OR Tonga OR Tuvalu OR Vanuatu OR Vietnam		
11j	25/2/ 2022	PubMed (Medline)	(((cyberbullying[**MeSH Terms**]) OR (cyberbullying[**MeSH Major Topic**])) **AND** ("Asia-Pacific" OR "South-asia" OR "East-asia" OR "Southeast asia" OR "South-east asia" OR "north-asia" OR oceania OR Australia OR Brunei OR Myanmar OR Cambodia OR China OR Fiji OR Indonesia OR Japan OR Kiribati OR Laos OR Malaysia OR "Marshall Islands" OR Micronesia OR Mongolia OR Nauru OR "New Zealand" OR "North Korea" OR Palau OR "Papua New guinea" OR Philippines OR Samoa OR Singapore OR "Solomon Islands" OR "South Korea" OR Taiwan OR Thailand OR "Timor-leste" OR Tonga OR Tuvalu OR Vanuatu OR Vietnam) AND (english[Filter] OR malay[Filter])) AND (("1995/1/1"[**Date - Publication**] : "2022/2/25"[**Date - Publication**]))	86	
11l			(((("cyberbullying/prevention and control"[**MeSH Terms**])) OR ("bullying/prevention and control"[**MeSH Terms**])) **AND** ("Asia-Pacific"[Text Word] OR "South-asia"[Text Word] OR "East-asia"[Text Word] OR "Southeast asia"[Text Word] OR "South-east asia"[Text Word] OR "north-asia"[Text Word] OR oceania[Text Word] OR Australia[Text Word] OR Brunei[Text Word] OR Myanmar[Text Word] OR Burma[Text Word] OR Cambodia[Text Word] OR China[Text Word] OR Fiji[Text Word] OR Indonesia[Text Word] OR Japan[Text Word] OR Kiribati[Text Word] OR Laos[Text Word] OR Malaysia[Text Word] OR "Marshall Islands"[Text Word] OR Micronesia[Text Word] OR Mongolia[Text Word] OR Nauru[Text Word] OR "New Zealand"[Text Word] OR "North Korea"[Text Word] OR Palau[Text Word] OR "Papua New guinea"[Text Word] OR Philippines[Text Word] OR Samoa[Text Word] OR Singapore[Text Word] OR "Solomon Islands"[Text Word] OR "South Korea"[Text Word] OR Taiwan[Text Word] OR Thailand[Text Word] OR "Timor-leste"[Text Word] OR Tonga[Text Word] OR	42	

			Tuvalu[Text Word] OR Vanuatu[Text Word] OR Vietnam[Text Word])) AND (("1995/1/1"[**Date - Publication**] : "2022/2/25"[**Date - Publication**]))		
12f	25/2/ 2022	Cambridge Journal Online	("electronic bully" OR "internet bully" OR "cyber abuse" OR "cyber harass" OR "cyberharass" OR "cyberthreat" OR "cyber threat" OR "cyberbully" OR "cyber bully" OR cyberstalk OR "cyber stalk" OR "cyberaggress" OR "cyber aggress" OR "cyber victim" OR "cybervictim" OR "Sextortion" OR "online bully" OR "bystander cyberbully" OR "cyber mobbing" OR "cybermobbing" OR "cyberbullying perpetration" OR "cyber violence" OR "digital bullying" OR "E-bullying" OR "online victimi?ation" OR "online violence" OR "online harassment" OR "online aggression" OR "phone bullying" OR "SMS bullying" OR "text bullying" OR "virtual aggression" OR "virtual mobbing" OR "internet harassment" OR "internet victimi?ation" OR "internet aggression" OR "online abuse") **AND** (intervention OR evaluate OR impact OR effectiveness OR prevention OR program OR effect OR efficacy)	39	• Content type: Articles
