# Supplementary material for: A systematic review and meta-analysis of interventions to decrease cyberbullying perpetration and victimization: An in-depth analysis within the Asia Pacific region
Source: Front Psychiatry. 2023 Jan 27;14:1014258. doi: 10.3389/fpsyt.2023.1014258 (PMC9911532; doi:10.3389/fpsyt.2023.1014258)
Supplement: Supplementary file 5 [file Data_Sheet_5.PDF]

Document 5

Summary of Studies in Full-Text Screening

id	eligible	reason for non-eligible	country	type	search	title	year	authors
786020195	y		China	Journal	Database Search	A pilot intervention study on bullying prevention among junior high school students in Shantou, China	2022	Peng, Z., Li, L., Su, X., & Lu, Y.
786020214	n	Not empirical study with comparative design	South Korea	Journal	Database Search	Can Online Education Programs Solve the Cyberbullying Problem? Educating South Korean Elementary Students in the COVID-19 Era	2021	Choi, E., & Park, N.
786020255	n	Not measuring CB perpetration/ victimization	Cyprus	Journal	Database Search	Improving Children's E-Safety Skills through an Interactive Learning Environment: A Quasi-Experimental Study	2020	Nicolaidou, I., & Venizelou, A.
786020257	n	Not in Asia-Pacific	Greece	Journal	Database Search	Raising awareness on cyber safety: adolescents' experience of a primary healthcare professional-led, school-based, multi-center intervention	2019	Tsimtsiou, Z., Drosos, E., Drontsos, A., Haidich, A. B., Dantsi, F., Sekeri, Z., ... & Arvanitidou, M.
786020258	n	Not in Asia-Pacific	Spain	Journal	Database Search	Effects of an incremental theory of personality intervention on the reciprocity between bullying and cyberbullying victimization and perpetration in adolescents	2019	Calvete, E., Orue, I., Fernández-González, L., & Prieto-Fidalgo, A.
786020286	n	Not in Asia-Pacific	Brazil	Journal	Database Search	Intervention in social skills and bullying	2018	Silva, J. L. D., Oliveira, W. A. D., Carlos, D. M., Lizzi, E. A.

								D. S., Rosário, R., & Silva, M. A. I.
786020293	n	Not in Asia-Pacific	United States	Journal	Database Search	A Mixed Methods Evaluation of the "Aged-Up" STAC Bullying Bystander Intervention for High School Students	2018	Johnston, A. D., Midgett, A., Doumas, D. M., & Moody, S.
786020299	y		Singapore	Journal	Database Search	iZ HERO adventure: Evaluating the effectiveness of a peer-mentoring and transmedia cyberwellness program for children	2017	Liau, A. K., Park, Y., Gentile, D. A., Katna, D. P., Tan, C. H. A., & Khoo, A.
786020304	y		Australia	Journal	Database Search	Longitudinal impact of the Cyber Friendly Schools program on adolescents' cyberbullying behavior	2016	Cross, D., Shaw, T., Hadwen, K., Cardoso, P., Slee, P., Roberts, C., Thomas, L., & Barnes, A
786020317	y		Taiwan	Journal	Database Search	Cyber Bullying Prevention: Intervention in Taiwan	2013	Lee, M. S., Zi-Pei, W., Svanström, L., & Dalal, K.
786020327	n	Not in Asia-Pacific	Belgium	Journal	Database Search	Thinking before posting? Reducing cyber harassment on social networking sites through a reflective message	2017	Van Royen, K., Poels, K., Vandebosch, H., & Adam, P.
786020359	n	No outcome data	Malaysia	Journal	Database Search	Development of a Personalized Mobile Mental Health Intervention for Workplace Cyberbullying Among Health Practitioners: Protocol for a Mixed Methods Study	2020	Kim, Y. J., Qian, L., & Aslam, M. S.
786020365	n	Not in Asia-Pacific	Germany	Journal	Database Search	Does the Information about Classroom Norms Change the Individual Injunctive Norms about Cyberbullying? A Minimal Intervention Study	2018	Pfetsch, J., Schultze-Krumbholz, A., & Füllgraf, F.

786020367	n	Not in Asia-Pacific	Germany	Journal	Database Search	Early childhood parenting and adolescent bullying behavior: Evidence from a randomized intervention at ten-year follow-up	2021	Kim, J. H., Hahlweg, K., & Schulz, W.
786020388	n	Review article	United States	Journal	Database Search	Instigators of Cyber-Bullying: A New Strategy for New Players	2019	Hicks, J., Clair, B. L., Waltz, M., Corvette, M., & Berry, S.
786020404	n	Not in Asia-Pacific	India	Journal	Database Search	Reducing Cyber-bullying and Problem Behaviors among Students through Parental Group Therapy	2016	Sandhu, D., & Kaur, S.
786020405	n	Not in Asia-Pacific	United States	Journal	Database Search	Reducing cyberbullying: A theory of reasoned action-based video prevention program for college students	2016	Doane, A. N., Kelley, M. L., & Pearson, M. R.
786020411	n	Not in Asia-Pacific	Spain	Journal	Database Search	Safety.Net: A Pilot Study on a Multi-Risk Internet Prevention Program	2021	Ortega-Barón, J., González-Cabrera, J., Machimbarrena, J. M., & Montiel, I.
786020418	y		Hong Kong	Journal	Database Search	Testing the Effectiveness of an E-Course to Combat Cyberbullying	2019	Leung, A. N. M., Wong, N., & Farver, J. M.
786020421	n	Not measuring CB perpetration/ victimization	Australia	Journal	Database Search	The Cyberbullying Picture Series (CyPicS): Developed for Use in Cyberbullying and Cyberbystander Research	2021	McLoughlin, L. T., Broadhouse, K. M., Clacy, A., Winks, N., Lagopoulos, J., & Hermens, D. F.
786020428	n	Review article	United States	Journal	Database Search	Using Solution-Focused Dramatic Empathy Training to Eliminate Cyber-Bullying	2016	Fröschle Hicks, J., Le Clair, B., & Berry, S.
786020484	n	Not measuring CB perpetration/ victimization	Malaysia	Journal	Database Search	The Use of Multimedia in Increasing Perceived Knowledge and Awareness of Cyber-bullying among Adolescents: A Pilot Study	2015	Wahab, N. A., Yahaya, W. A. J. W., & Muniandy, B.

786020493	n	Not in Asia-Pacific countries	Belgium	Journal	Database Search	The efficacy of the Friendly Attac serious digital game to promote prosocial bystander behavior in cyberbullying among young adolescents: A cluster-randomized controlled trial	2018	DeSmet, A., Bastiaensens, S., Van Cleemput, K., Poels, K., Vandebosch, H., Deboutte, G., ... & De Bourdeaudhuij, I.
786020532	n	Review article	Australia	Book Chapter	Database Search	Online social marketing approaches to inform cyber/bullying prevention and intervention: What have we learnt?	2018	Spears, B. A., Taddeo, C., & Barnes, A.
786020574	y		Thailand	Journal	Database Search	Effects of a formative assessment-based contextual gaming approach on studentsâ€™ digital citizenship behaviours, learning motivations, and perceptions	2020	Tapingkae, P., Panjaburee, P., Hwang, G. J., & Srisawasdi, N.
786020590	n	Not in Asia-Pacific	Hungary	Journal	Database Search	Gender-specific pathways regarding the outcomes of a cyberbullying youth education program	2022	Kapitány-Fövény, M., Lukács, J. Á., Takács, J., Kitzinger, I., Kiss, Z. S., Szabó, G., ... & Feith, H. J.
786020644	n	Not in Asia-Pacific	Netherland	Journal	Database Search	Empowering digital citizenship: An anti-cyberbullying intervention to increase children's intentions to intervene on behalf of the victim	2020	Vlaanderen, A., Bevelander, K. E., & Kleemans, M.
786020657	n	Not in Asia-Pacific	United States	Journal	Database Search	Feasibility of a Web-Accessible Game-Based Intervention Aimed at Improving Help Seeking and Coping Among Sexual and Gender Minority Youth: Results From a Randomized Controlled Trial	2021	Egan, J. E., Corey, S. L., Henderson, E. R., Abebe, K. Z., Louth-Marquez, W., Espelage, D., ... & Coulter, R. W.

786020661	n	Not in Asia-Pacific	Israel	Journal	Database Search	The Effectiveness of Safe Surfing Intervention Program in Reducing WhatsApp Cyberbullying and Improving Classroom Climate and Student Sense of Class Belonging in Elementary School	2021	Aizenkot, D., & Kashy-Rosenbaum, G.
786020665	n	No outcome data	South Korea	Journal	Database Search	Development of a Violence Prevention Educational Program for Elementary School Children Using Empathy (VPEP-E)	2020	Kang, S. R., Kim, S. J., & Lee, J.
786020666	n	Not empirical study with comparative design	Australia	Journal	Database Search	A Pilot Study of an Online Psychoeducational Program on Cyberbullying That Aims to Increase Confidence and Help-Seeking Behaviors among Adolescents	2020	Chillemi, K., Abbott, J. A. M., Austin, D. W., & Knowles, A.
786020669	y		Indonesia	Journal	Database Search	Effectiveness of solution-focused brief counseling to reduce online aggression of student	2020	Wiretna, C. D., Saputra, W. N. E., Muarifah, A., & Barida, M.
786020670	n	No outcome data	Australia	Journal	Database Search	The friendly schools initiative: Evidence-based bullying prevention in Australian schools	2019	Barnes, A., Pearce, N., Erceg, E., Runions, K., Cardoso, P., Lester, L., ... & Cross, D.
786020671	y		Australia	Journal	Database Search	Impact of the Friendly Schools whole-school intervention on transition to secondary school and adolescent bullying behaviour	2018	Cross, D., Shaw, T., Epstein, M., Pearce, N., Barnes, A., Burns, S., ... & Runions, K.
786020675	y		Australia	Book Chapter	Database Search	Cyber-Friendly Schools. Final report to Healthway	2018	Cross, D., Barnes, A., Cardoso, P., Hadwen, K., Shaw, T., Campbell, M., & Slee, P. T.

786020676	n	Not in Asia-Pacific	Netherland	Book Chapter	Database Search	Stop Online Bullies: The advantages and disadvantages of a standalone intervention	2018	Dehue, F., Völlink, T., Gunther, N., & Jacobs, N.
786020677	n	Not in Asia-Pacific	Spain	Journal	Database Search	Impact of cyberprogram 2.0 on different types of school violence and aggressiveness	2016	Garaigordobil, M. and MartÃnez-Valderrey, V.
786020680	n	Not in Asia-Pacific	Spain	Journal	Database Search	Knowing, building and living together on internet and social networks: The ConRed cyberbullying prevention program	2012	Ortega-Ruiz, R. and Del Rey, R. and Casas, J.A.
786020681	n	Not in Asia-Pacific	Italy	Journal	Database Search	Online and offline peer led models against bullying and cyberbullying	2012	Palladino, B. E., Nocentini, A., & Menesini, E.
786020738	n	Not empirical study with comparative design	Taiwan	Journal	Database Search	"Think before You Type": The Effectiveness of Implementing an Anti-Cyberbullying Project in an EFL Classroom	2018	Chen, C. W. Y.
786020761	n	Not empirical study with comparative design	Australia	Journal	Database Search	If It's about Me, Why Do It without Me? Genuine Student Engagement in School Cyberbullying Education	2015	Cross, D., Lester, L., Barnes, A., Cardoso, P., & Hadwen, K.
786020801	n	Not measuring CB perpetration/ victimization	Indonesia	Journal	Database Search	The development and pilot testing of an adolescent bullying intervention in Indonesia–the ROOTS Indonesia program	2019	Bowes, L., Aryani, F., Ohan, F., Haryanti, R. H., Winarna, S., Arsianto, Y., ... & Minnick, E.
786020815	n	Not measuring CB perpetration/ victimization	Thailand	Journal	Database Search	Social Intelligence Counseling Intervention to Reduce Bullying Behaviors Among Thai Lower Secondary School Students: A Mixed-method Study	2021	Jueajinda, S., Stiramon, O., & Ekpanyaskul, C.
786020816	n	Not measuring CB	South Korea	Journal	Database Search	Effects of a smartphone application for cognitive	2019	Kang, J., & Jeong, Y. J.

		perpetration/ victimization				rehearsal intervention on workplace bullying and turnover intention among nurses		
786020817	n	Not measuring CB perpetration/ victimization	Australia	Journal	Database Search	A Novel Approach to Tackling Bullying in Schools: Personality-Targeted Intervention for Adolescent Victims and Bullies in Australia	2020	Kelly, E. V., Newton, N. C., Stapinski, L. A., Conrod, P. J., Barrett, E. L., Champion, K. E., & Teesson, M.
786020825	n	Not measuring CB perpetration/ victimization	Australia	Journal	Database Search	Developing wellbeing through a randomised controlled trial of a martial arts based intervention: An alternative to the anti-bullying approach	2019	Moore, B., Woodcock, S., & Dudley, D.
786020826	n	Not measuring CB perpetration/ victimization	Taiwan	Journal	Database Search	Effects of a Collaborative Board Game on Bullying Intervention: A Group-Randomized Controlled Trial	2018	Nieh, H. P., & Wu, W. C.
786020830	n	Not CB intervention	Australia	Journal	Database Search	How teachers deal with cases of bullying at school: What victims say	2020	Rigby, K.
786020854	n	Not measuring CB perpetration/ victimization	Australia	Journal	Database Search	Coping in the Cyberworld: Program Implementation and Evaluation–A Pilot Project	2009	Lam, C. W. C., & Frydenberg, E.
786020865	n	Not empirical study with comparative design	Australia	Journal	Database Search	Cyberbullying prevention: One primary schools approach	2010	Tangen, D., & Campbell, M.
786020866	n	Not empirical study with comparative design	Australia	Journal	Database Search	A cyberbullying intervention with primary-aged students	2012	Toshack, T., & Colmar, S.

786020877	n	Not in Asia-Pacific	United States	Journal	Database Search	Assessing the effects of the dating violence prevention program "safe dates" using random coefficient regression modeling	2005	Foshee, V. A., Bauman, K. E., Ennett, S. T., Suchindran, C., Benefield, T., & Linder, G. F.
786020880	n	Not in Asia-Pacific	United States	Journal	Database Search	Efficacy of a randomized trial of a community and school-based anti-violence media intervention among small-town middle school youth	2008	Swaim, R. C., & Kelly, K.
786020897	n	Not in Asia-Pacific	United States	Journal	Database Search	Engagement Matters: Lessons from Assessing Classroom Implementation of Steps to Respect: A Bullying Prevention Program Over a One-year Period	2014	Low, S., Van Ryzin, M. J., Brown, E. C., Smith, B. H., & Haggerty, K. P.
786020908	n	Not in Asia-Pacific	United States	Journal	Database Search	The Effects of Moms and Teens for Safe Dates: A Dating Abuse Prevention Program for Adolescents Exposed to Domestic Violence	2015	Foshee, V. A., Benefield, T., Dixon, K. S., Chang, L. Y., Senkomago, V., Ennett, S. T., ... & Bowling, J. M.
786020924	n	Not in Asia-Pacific	United States	Journal	Database Search	Preventing Adolescent Social Anxiety and Depression and Reducing Peer Victimization: Intervention Development and Open Trial	2016	La Greca, A. M., Ehrenreich-May, J., Mufson, L., & Chan, S.
786020934	n	Not measuring CB perpetration/ victimization	Australia	Journal	Database Search	Family Involvement in a Whole-School Bullying Intervention: Mothers' and Fathers' Communication and Influence with Children	2017	Lester, L., Pearce, N., Waters, S., Barnes, A., Beatty, S., & Cross, D.
786020941	n	Not in Asia-Pacific	United States	Journal	Database Search	Project Date SMART: a Dating Violence (DV) and Sexual Risk Prevention Program for	2018	Rizzo, C. J., Joppa, M., Barker, D., Collibee, C., Zlotnick, C., & Brown, L. K.

						Adolescent Girls with Prior DV Exposure		
786020942	n	Not in Asia-Pacific	Netherland	Journal	Database Search	Prevention of violent revictimization in depressed patients with an add-on internet-based emotion regulation training (iERT): Study protocol for a multicenter randomized controlled trial	2018	Christ, C., de Waal, M. M., van Schaik, D. J., Kikkert, M. J., Blankers, M., Bockting, C. L., ... & Dekker, J. J.
786020947	n	Not in Asia-Pacific	Netherland	Journal	Database Search	BEATVIC, a body-oriented resilience therapy using kickboxing exercises for people with a psychotic disorder: A feasibility study	2018	de Vries, B., van der Stouwe, E. C., Waarheid, C. O., Poel, S. H., van der Helm, E. M., Aleman, A., ... & van Busschbach, J. T.
786020950	n	Not in Asia-Pacific	United States	Journal	Database Search	Bystander Program Effectiveness to Reduce Violence Acceptance: RCT in High Schools	2019	Coker, A. L., Bush, H. M., Brancato, C. J., Clear, E. R., & Recktenwald, E. A. (
786020951	n	Not in Asia-Pacific	United States	Journal	Database Search	Coaching Teachers to Detect, Prevent, and Respond to Bullying Using Mixed Reality Simulation: an Efficacy Study in Middle Schools	2019	Pas, E. T., Waasdorp, T. E., & Bradshaw, C. P.
786020952	n	Not in Asia-Pacific	United States	Journal	Database Search	Evaluation of a Bystander-Focused Interpersonal Violence Prevention Program with High School Students	2019	Edwards, K. M., Banyard, V. L., Sessarego, S. N., Waterman, E. A., Mitchell, K. J., & Chang, H.
786020953	n	Not in Asia-Pacific	United States	Journal	Database Search	Evaluation of a Whole-School Change Intervention: Findings from a Two-Year Cluster-Randomized Trial of the Restorative Practices Intervention	2019	Acosta, J., Chinman, M., Ebener, P., Malone, P. S., Phillips, A., & Wilks, A.

786020971	n	Not in Asia-Pacific	United States	Journal	Database Search	The Effects of Practitioner-Delivered School-Based Mental Health on Aggression and Violence Victimization in Middle Schoolers	2020	Morgan-Lopez, A. A., Saavedra, L. M., Yaros, A. C., Trudeau, J. V., & Buben, A.
786020975	n	Not in Asia-Pacific	United States	Journal	Database Search	Creating Supportive Contexts for Early Adolescents during the First Year of Middle School: Impact of a Developmentally Responsive Multi-Component Intervention	2020	Dawes, M., Farmer, T., Hamm, J., Lee, D., Norwalk, K., Sterrett, B., & Lambert, K.
786020987	n	Not in Asia-Pacific	Netherland	Journal	Database Search	Neural changes following a body-oriented resilience therapy with elements of kickboxing for individuals with a psychotic disorder: a randomized controlled trial	2021	van der Stouwe, E. C., Pijnenborg, G. H., Opmeer, E. M., de Vries, B., Marsman, J. B. C., Aleman, A., & van Busschbach, J. T.
786020999	n	Not in Asia-Pacific	United States	Journal	Database Search	The Preventing Relational Aggression in Schools Everyday (PRAISE) Program: Adaptations to Overcome Subgroup Differences in Program Benefits	2022	Waasdorp, T. E., Paskewich, B. S., Waanders, C., Fu, R., & Leff, S. S.
786021014	n	Not in Asia-Pacific	United States	Journal	Database Search	Impact of the RIPP Violence Prevention Program on Rural Middle School Students	2003	Farrell, A. D., Valois, R. F., Meyer, A. L., & Tidwell, R. P.
786021050	n	Not in Asia-Pacific	United States	Journal	Database Search	The Bullying Literature Project: Using Children's Literature to Promote Prosocial Behavior and Social-Emotional Outcomes Among Elementary School Students	2015	Wang, C., Couch, L., Rodriguez, G. R., & Lee, C.
786021113	n	Not in Asia-Pacific	Germany	Journal	Database Search	Prevention 2.0: Targeting Cyberbullying @ School	2014	Wölfer, R., Schultze-Krumbholz, A., Zagorscak, P.,

								Jäkel, A., Göbel, K., & Scheithauer, H.
786021142	y		Hong Kong	Journal	Database Search	A Cyberbullying Intervention for Hong Kong Chinese College Students	2018	Leung, A. N. M., Fung, D. C. L., & Farver, J. M.
786021153	n	Not in Asia-Pacific	United States	Journal	Database Search	Bystanders Against Cyberbullying: a Video Program for College Students	2020	Doane, A. N., Ehlke, S., & Kelley, M. L. (
786021172	y		Malaysia	Thesis	Other Source	Brief Mindfulness Practice as an Intervention on the Relationship between Cyberbullying and Depressive Symptom among Young Adults in Malaysia	2019	Choo, B. B. C., Lee, Z., & Ng, J. Q.
786021173	n	No outcome data	Malaysia	Journal	Other Source	Examining the efficacy of the Olweus prevention programme in reducing bullying: the Malaysian experience	2009	Yaakub, N. F., Haron, F., & Leong, G. C.
